# Social and Environmental Determinants Show Greater Predictive Attribution than Health Behaviours for Multimorbidity: A Population-Based Machine Learning Study

**DOI:** 10.3390/healthcare14213565

**Published:** 2026-10-22

**Authors:** Rodrigo Yáñez-Sepúlveda, Alejandra Uribe-Díaz, Rodrigo Olivares, José Francisco Tornero-Aguilera, Vicente Javier Clemente-Suárez, Alexandra Martín-Rodríguez, Josivaldo de Souza-Lima, Camila Miño, Fiorella Quiroz-Cárdenas, José Francisco López-Gil

**Affiliations:** 1 School of Sports Science, Faculty of Education and Social Sciences, Universidad Andrés Bello, Viña del Mar 2520000, Chile; rodrigo.yanez.s@unab.cl (R.Y.-S.); josivaldo.desouza@unab.cl (J.d.S.-L.); 2 Department of Sport Sciences, Faculty of Sport and Health Sciences, Fit Generation Research Institute, AD500 Andorra la Vella, Andorra; jtornero@fitgeneration.es; 3 Faculty of Health Sciences, Universidad Tecnológica Atlántico Mediterráneo—UTAMED, 29590 Málaga, Spain; alejandra.uribe21@gmail.com (A.U.-D.); vctxente@yahoo.es (V.J.C.-S.); 4 Department of Health Research, Icen Cognis, 38370 Tenerife, Spain; 5 School of Computer Engineering, Universidad de Valparaíso, Valparaíso 2340000, Chile; rodrigo.olivares@uv.cl; 6 Grupo de Investigación ACTIVE, Facultad de Ciencias de la Educación, UNIE Universidad, 28015 Madrid, Spain; sandra.martin.rodriguez8@gmail.com; 7 Universidad Internacional Para el Desarrollo (UNINDE), 06001 Badajoz, Spain; 8 Vicerrectoría de Investigación y Postgrado, Universidad de Los Lagos, Osorno 5290000, Chile; fiorella.quiroz@yahoo.com; 9 School of Medicine, Universidad Espíritu Santo, Samborondón 092301, Ecuador; 10 Faculty of Health Sciences, Universidad Autónoma de Chile, Santiago 7500000, Chile

**Keywords:** multimorbidity, social determinants of health, machine learning, SHAP, complex survey design, neighbourhood environment, social isolation, health behaviours, Chile, ENCAVI

## Abstract

**Background/Objectives**: Multimorbidity is usually attributed to ageing and health behaviours; social and environmental exposures are rarely quantified in the same model. We estimated how much of the predictive attribution for chronic condition burden, beyond age, falls to social, environmental, and behavioural domains. **Methods**: We analysed 16,590 respondents aged ≥15 years in the 2023–2024 Chilean National Quality of Life and Health Survey, counting 19 physician-diagnosed chronic conditions. After removing age with design-weighted splines, we modelled the residual count on 151 upstream determinants with 17 supervised algorithms, using survey weights and disjoint sampling units for training and testing. SHapley Additive exPlanations (SHAP) values were aggregated into pre-specified domains, summed, and averaged per variable. **Results**: Among 12,070 complete cases, weighted multimorbidity prevalence was 40.1%; age alone explained 23.2% of variance. The random forest used for attribution reached test R^2^ = 0.117 (95% confidence interval 0.091–0.138), leaving 88.3% of residual variation unexplained. Predictive attribution was highest for social isolation and support (23.1%), neighbourhood environment (20.9%), and socio-economic position (20.3%), whereas health behaviours received 12.0% and the lowest attribution per variable of any substantive domain. Each of these domains exceeded health behaviours with age modelled directly, with size-normalised or grouped attribution, with reweighting for exclusion, and without mental health diagnoses in the count; among adults aged 15–44 years, however, health behaviours received the largest summed attribution (20.9%). **Conclusions**: After accounting for age, social isolation, neighbourhood conditions, and material deprivation received greater predictive attribution within the final model than health behaviours did, whereas behaviours led among younger adults, suggesting age-specific pathways into multimorbidity. Attribution is not a causal estimate; most variation remained unexplained, and reverse causation cannot be excluded. Nevertheless, the ordering questions behaviour-centred framings of prevention, and these domains warrant evaluation as prevention targets in longitudinal and intervention studies.

## 1. Introduction

Multimorbidity, the co-occurrence of two or more chronic conditions in the same person, has become the normal presentation of chronic disease rather than an exception. Community-based estimates place its global prevalence at roughly one-third of the adult population [1], and pooled estimates for Latin America and the Caribbean are of comparable magnitude [2]. The conditions that constitute it are leading contributors to years lived with disability worldwide [3], and it is expressed across essentially every organ system, from heart failure [4] and stroke [5] to chronic obstructive pulmonary disease (COPD) [6]. Health systems organised around single-disease pathways manage it poorly: overviews of intervention studies find limited evidence that disease-specific approaches improve outcomes when several conditions coexist [7].

The dominant explanatory framework attributes multimorbidity to two forces: chronological ageing and individual health behaviours. Ageing is unquestionably central, and the accumulation of conditions across the life course is among the most reproducible findings in chronic disease epidemiology [8]; the intrinsic capacity framework has formalised this trajectory and shown its predictive value for later outcomes [9]. The behavioural component also has an extensive evidence base, with sedentary time, inactivity, diet, alcohol, and sleep each linked to incident conditions and to mortality, and with lifestyle factors predicting incident multimorbidity in prospective cohorts [10,11]. Prevention policy in most middle-income countries, Chile included, is organised primarily around this second component.

A parallel body of literature points elsewhere. Socio-economic deprivation is associated with earlier onset and greater accumulation of conditions [12], and with worse short-term survival once chronic disease is established [13]; reviews of the social determinants of chronic disease describe education, income, occupation, and neighbourhood as structural causes rather than background variables [14]. Social isolation and loneliness carry effect sizes comparable to established behavioural risks [15], place of residence is itself associated with multimorbidity [16], and violence and fear of crime are associated with worse health [17]. These domains, however, are almost always studied one at a time, in separate studies and separate models. As a result, their weight relative to one another, and relative to behaviours, has not been estimated within a common model; what such an estimate can deliver is a ranking of predictive contributions rather than a ranking of causes.

Quantifying relative predictive contribution requires three methodological commitments that are often absent. First, age must be removed flexibly rather than adjusted linearly, because its relationship with condition counts is markedly non-linear; otherwise, residual age effects are attributed to correlated social exposures. Second, the analysis must accommodate many candidate exposures whose effects may be non-linear and interactive. This is where supervised machine learning enters the design, and its role should be stated plainly: the aim of this study was not to apply machine learning but to test a hypothesis, and machine learning was the instrument that made the test possible. A single model, such as a generalised linear model with a least absolute shrinkage and selection operator (LASSO) penalty, would build the assumption that every determinant acts additively and linearly into the answer; if that assumption were wrong, the ranking of domains would be an artefact of the model rather than a property of the data, and a single model cannot reveal whether it is wrong. Because no algorithm can be shown in advance to be best for a given dataset [18,19], and because flexible algorithms often fail to improve on well-specified regression in health data [20], the presence of non-linear structures is an empirical question that has to be settled by comparison. We therefore pre-specified a panel spanning the main families of supervised regression, with the selection rule fixed before any model was fitted: the gap between the best flexible and the best linear model measures how much of a non-linear structure the determinants carry, and fixing the panel and rule in advance removes the choice of algorithm as a researcher’s degree of freedom. We also report the attribution obtained from a tuned penalised linear model alongside that of the selected model, so that readers can judge whether the ordering depends on the algorithm. The specific methods and their known pitfalls are described in Section 2. Third, when data come from a complex national survey, calibrated weights and the clustered design must be respected in both estimation and validation, since ignoring them biases point estimates and produces optimistic performance through leakage between observations sharing a sampling unit.

A further requirement is specific to this question and is frequently overlooked. Multimorbidity has consequences (i.e., reduced mobility, low energy, poor self-rated health, health service use, and body dissatisfaction) that are measured in the same surveys as its determinants. Including such variables produces models that appear to perform well while explaining nothing: they recover the shadow of the disease rather than its antecedents. Any credible attribution analysis must therefore exclude downstream variables explicitly and state the exclusion rule.

We applied these principles to the 2023–2024 Chilean National Quality of Life and Health Survey. Chile is an informative setting: an upper-middle-income country with a rapidly ageing population, high chronic disease burden, and pronounced socio-economic and territorial inequalities, in which multimorbidity has been documented in national health survey data [21], and a substantial share of mortality is attributable to sitting time and physical inactivity [22]. Our hypothesis, formulated before any model was fitted, was that once age had been removed, the social and environmental domains would receive at least as much predictive attribution as health behaviours. Our objectives followed from it and were threefold. First, we estimated how much of the predictive attribution for the chronic condition count, after removing age, falls to each of eight pre-specified domains: social isolation and support, neighbourhood environment, socio-economic position, demographics, health behaviours, violence and discrimination, mobility and transport, and a residual “other” group of household composition and area variables. Second, we identified the strongest individual determinants within those domains. Third, we tested whether the resulting ordering is reproducible across independent partitions of the data and robust to the handling of age and to the exclusion of incomplete cases. Five of these domains carry the substantive contrast of interest; demographics, mobility, and the residual group are retained so that attribution sums to the whole model rather than to a selected subset. Throughout, “predictive attribution” denotes the share of a fitted model’s predictions that the attribution method assigns to a variable or domain. It is a descriptive quantity within that model and not an estimate of causal effect.

## 2. Materials and Methods

### 2.1. Study Design and Data Source

We conducted a cross-sectional secondary analysis of the 2023–2024 Chilean National Quality of Life and Health Survey (Encuesta Nacional de Calidad de Vida y Salud, ENCAVI), a population-based survey of people aged 15 years or older using stratified multistage sampling with unequal selection probabilities. The dataset comprised 16,590 respondents and 464 variables. Population estimation used the calibrated person weight, which is constructed on four margins (sex by age, sex by education, region, and macro-area), together with the stratum identifier (96 strata) and primary sampling unit (PSU) identifier (350 PSUs). Ignoring these design components biases both point estimates and their uncertainty when selection probabilities are informative [23]. We followed the Strengthening the Reporting of Observational Studies in Epidemiology (STROBE) recommendations.

### 2.2. Outcome

The outcome was the number of chronic conditions reported, as diagnosed by a physician, counted across 19 individual survey items: hypertension, diabetes, stroke, COPD or asthma, liver disease, epilepsy, mental health disorders, autism spectrum disorder, chronic kidney disease, eye disease, joint disease, digestive disease, cardiac insufficiency or arrhythmia, urinary incontinence, chronic pain, hearing loss, vision loss, myocardial infarction, and other chronic conditions. Responses of “does not know” or “does not answer” were treated as missing, and respondents missing more than two items were excluded. The epilepsy item is administered only to a filtered subgroup and was coded as absent when not applicable. We used the count rather than the survey-provided three-level categorisation, which is top-coded at “two or more” and discards the upper tail. Multimorbidity was defined as two or more conditions.

### 2.3. Candidate Determinants and Exclusion of Downstream Variables

**Figure 1:**
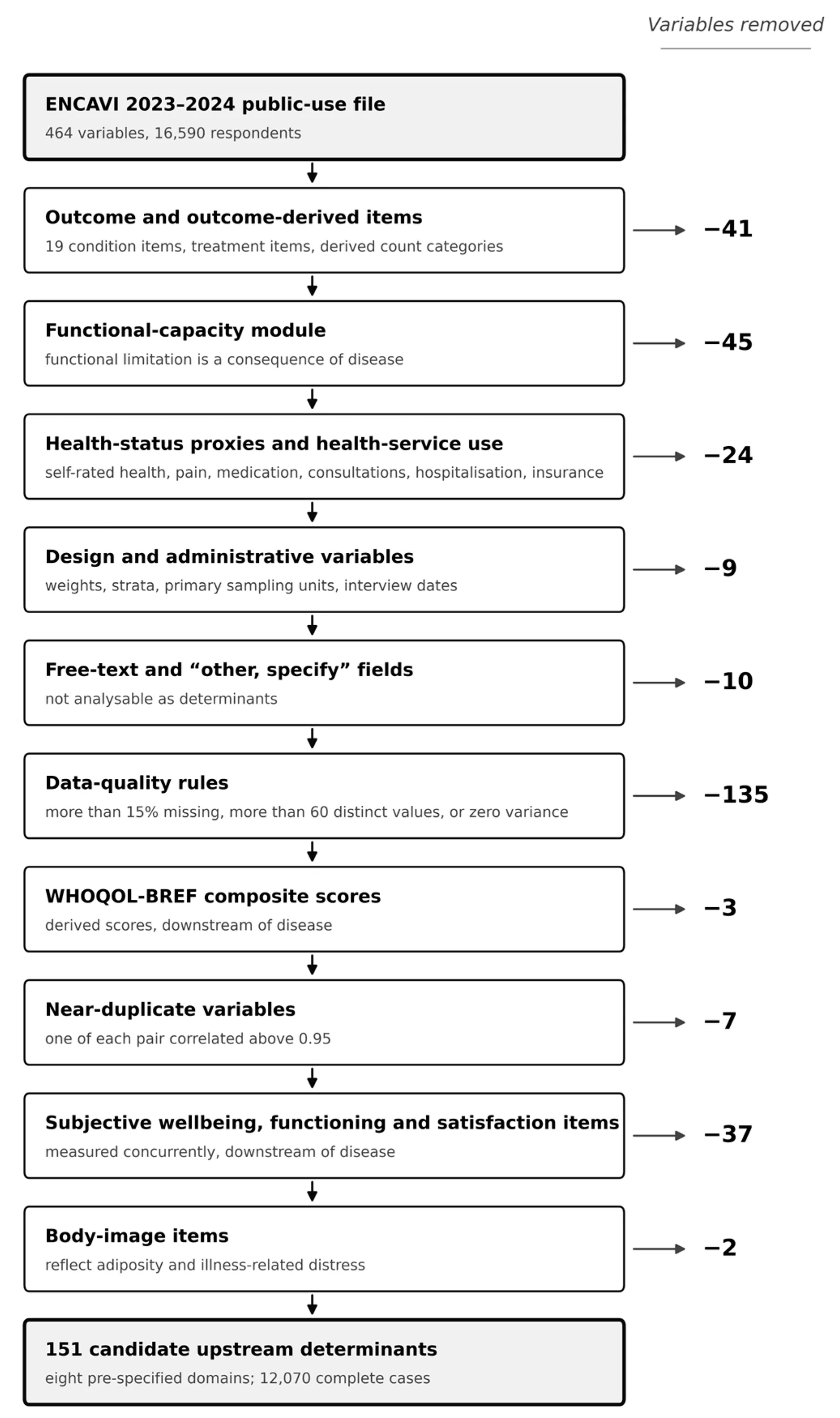
Sequence used to reduce the 464 survey variables to 151 candidate upstream determinants. Each box gives the rule applied, and the right-hand column shows the number of variables it removed. The rule is deliberately conservative: any variable plausibly measuring a consequence of chronic disease was excluded, even at the cost of discarding informative predictors.

From the 464 available variables, we retained candidate determinants using an explicit, pre-specified exclusion sequence. Figure 1 shows the operational order. We first removed all variables entering the outcome definition, including the individual condition items, treatment-for-condition items, and the derived condition count categorisation. We then removed the entire functional capacity module, since functional limitation is a consequence of chronic disease rather than an antecedent. We next removed 24 variables identified by keyword screening as health status proxies or health service use: self-rated health, satisfaction with health, pain interference, medication use, medical consultations, hospitalisation, and health insurance affiliation. Survey design and administrative variables (weights, strata, primary sampling unit identifiers, and interview dates; 9 variables) were removed next, followed by free-text and “other, specify” fields (10 variables); neither set was eligible as determinants. Among the variables still remaining, those with more than 15% missingness, more than 60 distinct values, or zero variance were dropped. The World Health Organization Quality of Life-BREF (WHOQOL-BREF) composite scores were removed next, because these derived scores are downstream of disease. One member of each pair correlated above 0.95 was then removed to avoid duplicated information. We then removed the subjective wellbeing, functioning, and satisfaction items, because these are measured concurrently and are strongly downstream of disease. Finally, we removed two body-image items, which in preliminary models ranked first but are best understood as reflections of adiposity and of illness-related distress rather than determinants.

The analytic sample was restricted to respondents with a complete outcome and complete data on a core set of demographic and behavioural indicators (age, sex, education, physical activity category, moderate-to-vigorous physical activity, walking, sitting time, sleep duration, alcohol use, and body mass index), so that the behavioural block was fully observed; this is the only complete-case step. Sitting time is recorded in the survey in two fields, hours and minutes per day, which entered the determinant set as two separate items (Table S2); descriptive statistics (Section 3.1) express it in hours per day. Non-response codes (7, 8, 9, 77, 88, 99, and their three- and four-digit equivalents) were recoded as missing wherever they accounted for less than 35% of an item’s responses, including in binary items, where codes such as 9 or 99 would otherwise be read as valid responses. Because this rule also treats some valid answers as missing in items whose genuine categories reach 7 to 9 (for example, widowed marital status, seven days of activity, or eight hours of sitting), Section 2.7 repeats the analysis with a stricter rule. The remaining missing values were imputed at the variable median. This yielded 151 candidate determinants spanning demographics, socio-economic position, work and occupation, health behaviours, social isolation and support, neighbourhood physical and social environment, violence and discrimination, and mobility. The number of variables removed at each step is given in Figure 1 and, with the reason for each step, in Table S1.

Median imputation was used rather than multiple imputation for three reasons. First, the exclusion sequence had already removed every variable with more than 15% missingness, so only 1.6% of the cells of the analytic matrix were imputed: 16 of the 151 determinants had no missing values at all, the median determinant had 0.6% missing, and only 13 exceeded 5%, the highest being the household heating fuel item at 13.9%. Second, multiple imputation would require the attribution step to be repeated in every imputed dataset and the results to be pooled, and no established rule exists for pooling Shapley attributions across imputations; averaging them would conceal between-imputation variability in the ranking rather than represent it. Third, the concern that median imputation distorts the importance ranking is testable, and Section 2.7 tests it rather than assuming it away. Missingness was not uniform across domains, being highest in the socio-economic (3.2% of cells) and behavioural (3.1%) blocks and lowest in violence and discrimination (0.3%), which is itself a reason to check its influence.

### 2.4. Two-Stage Age Adjustment

**Figure 2:**
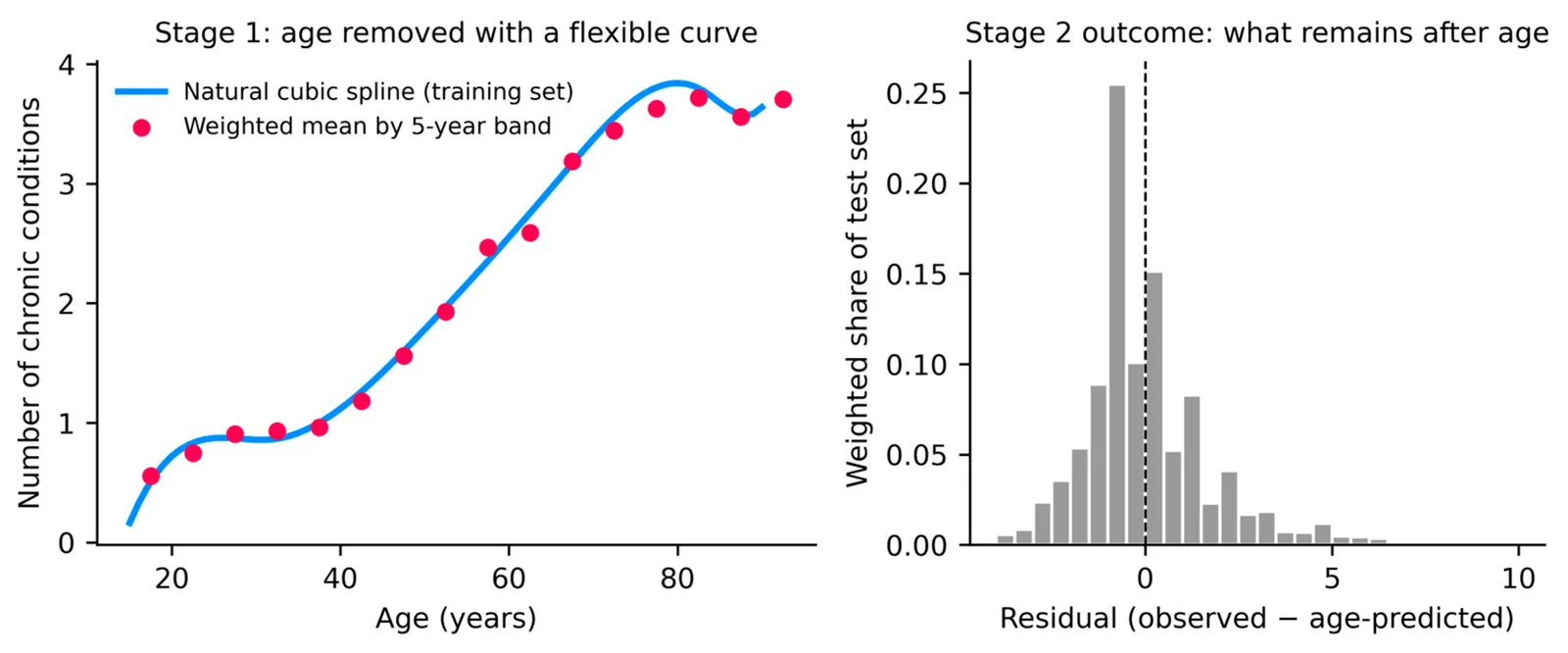
The two-stage handling of age. (**Left**): design-weighted mean number of chronic conditions by five-year age band (points) and the natural cubic spline fitted in the training set (line); this first stage accounts for 23.2% of the variance in the test set. (**Right**): distribution of the residual after removing the spline fit, which is the outcome modelled in the second stage; its weighted standard deviation in the test set is 1.69 conditions. The dashed vertical line marks a residual of zero, i.e., an observed number of chronic conditions equal to the age-predicted value; positive residuals indicate more conditions than expected for age, and negative residuals fewer. The second-stage model can only recover variation that remains after this step.

Because age dominates chronic condition accumulation and is correlated with most social exposures, we removed it in a separate first stage rather than including it as one predictor among many. Natural cubic splines of age (degree 3, six knots, linear extrapolation) were fitted to the condition count using design-weighted linear regression in the training set, and the analysis outcome for the second stage was the residual from this fit. This ensures that the second-stage model cannot recover variation attributable to age and makes the reported quantities interpretable as contributions over and above age. The age band was nonetheless retained among the candidate determinants, so that any residual age structure would be visible rather than silently redistributed. Figure 2 illustrates the two stages with the study data. Removing age first answers a specific question, namely how the remaining determinants relate to condition counts among people of the same age; it does not estimate the contribution of age itself, which is reported separately from the first stage. Because this choice could in principle alter the apparent contribution of age-related social exposures, Section 2.7 describes a sensitivity analysis in which age is not removed but modelled directly as a predictor.

### 2.5. Supervised Modelling and Validation

We compared 17 supervised regression algorithms: linear regression, ridge, LASSO, elastic net, k-nearest neighbours, support vector regression (SVR) with a radial basis function (RBF) kernel, decision tree, random forest, extremely randomised trees (Extra Trees), gradient boosting, histogram gradient boosting, adaptive boosting (AdaBoost), bagging, multilayer perceptron, light gradient-boosting machine (LightGBM), extreme gradient boosting (XGBoost), and categorical boosting (CatBoost). The panel was assembled to cover every major family of supervised regression rather than to reach a particular number: four linear models (unpenalised, ridge, LASSO, and elastic net), a kernel method (SVR), an instance-based method (k-nearest neighbours), a single decision tree, three averaging ensembles of randomised trees (random forest, Extra Trees, and bagging of trees), six boosting ensembles (gradient boosting, histogram gradient boosting, AdaBoost, LightGBM, XGBoost, and CatBoost), and a neural network (multilayer perceptron). The number follows from that coverage: the panel comprises the commonly used regression estimators of these families in scikit-learn, together with the three gradient-boosting libraries in widest use, and no candidate was added or removed after results were seen. The panel answers two questions that a single model cannot. First, whether the determinants act non-linearly and interactively can only be established by comparing families that differ in that capacity; a generalised linear model with a LASSO penalty on its own would have assumed the answer rather than tested it. Second, choosing one algorithm before seeing its performance would risk a conclusion that depends on that choice, whereas choosing it afterwards would be a researcher’s degree of freedom; a pre-specified panel with a fixed selection rule, the highest cross-validated R^2^, avoids both. Had a linear model performed best, attribution would have been computed from its coefficients; that attribution is reported for the tuned elastic net in any case (Section 3.7), as a check that the domain ordering does not depend on the use of a tree ensemble. Comparable supervised panels have been applied to cardiovascular events in diabetes and chronic kidney disease [24] and depression risk in COPD [25]; established risk equations illustrate both the value and the limits of prediction in this space [26,27]. Data were divided into training (70%) and test (30%) sets using grouped random splitting on the primary sampling unit, so that no PSU contributed observations to both sets; this prevents the optimistic bias that arises when respondents from the same cluster appear in training and test sets. Model selection used five-fold cross-validation with the same PSU grouping. Calibrated survey weights were supplied to the fitting procedure for every estimator that accepts observation weights, and all performance metrics were weighted. The settings of the 17-algorithm panel were fixed rather than searched: the random forest and Extra Trees used 300 trees, a minimum leaf size of 20, and one-third of the determinants at each split, and the settings of the other algorithms are listed in the deposited code. Because fixed settings can handicap some families and penalised linear models in particular, we also tuned the three penalised linear models and five tree ensembles using nested cross-validation (Table S14). Within each of the five PSU-grouped outer folds of the training set, a three-fold PSU-grouped inner search selected the hyperparameters, and the outer fold scored the selected model, so that no observation used for scoring was used for selection. The domain attribution was then repeated with the best-tuned model, with the tuned random forest, and with the tuned elastic net, for which the attribution of each determinant is its coefficient multiplied by its centred value. Performance was summarised by R^2^, mean absolute error (MAE), and root mean squared error (RMSE), each referenced explicitly to a null model predicting the weighted mean. All algorithms were run in Python 3.12: fourteen were taken from the scikit-learn library; LightGBM, XGBoost, and CatBoost were taken from their own libraries; and attribution used the shap library. The module used for each algorithm is reported with the performance results (Section 3.3); the versions used were Python 3.12.3 (Python Software Foundation, Wilmington, DE, USA), scikit-learn 1.8.0, LightGBM 4.7.0, XGBoost 3.4.1, CatBoost 1.2.10, shap 0.52.0, numpy 2.4.4, pandas 3.0.2, scipy 1.17.1, and pyreadstat 1.3.6, and they are pinned in the repository.

### 2.6. Attribution and Robustness

SHapley Additive exPlanations (SHAP) values were computed for the selected model on the test set using TreeExplainer, and the mean absolute SHAP value was used as the attribution measure for each determinant, following current practical guidance on SHAP analysis [28]. In plain terms, a SHAP value states how much one variable moved the model’s prediction for one respondent away from the average prediction; averaging its absolute value over respondents gives the weight of that variable in the fitted model. This choice is deliberate: impurity-based random forest importance is frequently misused in medical applications and is biased towards continuous, high-cardinality predictors [29], whereas correctly applied variable-importance methods have supported environmental attribution analyses in population health [30]. Determinants were aggregated by variable prefix into the eight pre-specified domains defined in Section 1, and domain attribution was defined as the sum of member attributions. The prefix-to-domain mapping was fixed in the analysis configuration file, which is deposited with the code, before any model was fitted. This study was not prospectively registered. Mean absolute SHAP values were averaged over test-set respondents without survey weights; Table S13 repeats the domain shares with weighted averages. Because summing mean absolute SHAP values over correlated members does not give an additive decomposition, domain shares are descriptive. We therefore also computed two grouped measures (Table S13): a grouped SHAP attribution, in which the signed SHAP values of a domain’s members are summed for each respondent before the absolute value is taken and averaged, so that opposing contributions within a domain cancel; and a grouped permutation importance, the loss of weighted test R^2^ when all members of a domain are permuted jointly, averaged over 20 permutations. Because the domains contain between 5 and 45 variables, and a domain with more members has more opportunity to accumulate attribution, we report the summed attribution together with the mean attribution per variable, and we repeat the comparison under two further normalisations in Table S10: the median attribution of a domain’s members, and the sum restricted to the five leading variables of each domain, with five being the size of the smallest domain. A domain that led only because it is large would fall under these normalisations. The direction of each association was obtained from the correlation between the variable and its SHAP values. Robustness was assessed in three ways: repetition of the entire pipeline across five independent PSU-grouped splits with different seeds; bootstrap confidence intervals for all test metrics from 1000 resamples; and Spearman correlation of the importance ranking across seeds. All analyses used Python 3.12, with a fixed random seed of 42.

### 2.7. Sensitivity Analyses

Several sensitivity analyses tested whether the domain ordering depends on analytical choices. First, we repeated the supervised step on principal components extracted from the same determinants to test whether dimensionality reduction improved performance. Second, we removed the age residualisation entirely: the raw condition count was the outcome, continuous age (standardised) was added to the 151 determinants alongside age band, and the same PSU-grouped split, random forest, and SHAP attribution were applied. Domain shares were computed with the two age variables assigned to demographics and, to allow comparison with the main analysis, also after excluding them and renormalising the remaining attribution. Third, we refitted the age-residualised model without the age band being among the predictors, to check whether its retention altered the attribution of the other domains. Fourth, we examined the complete-case exclusion. Included and excluded respondents were compared on demographic, socio-economic, social, and environmental characteristics, and on the outcome where it was observed (Table S7). A design-weighted logistic regression of inclusion on age, sex, education, income quintile, urban–rural residence, and region was then fitted; the survey weight was multiplied by the inverse of the fitted inclusion probability; and the age residualisation, random forest fit, and attribution were repeated with these weights on the same split. Fifth, we tested the influence of median imputation in two ways: by refitting after dropping the 13 determinants with more than 5% missingness, and by refitting with a binary missingness indicator added for each of those 13 variables, so that the model could use the fact that a value had been imputed. Sixth, to address reverse causation and to examine whether the determinants of early chronic disease differ from those of disease accumulated over a lifetime, we repeated the pipeline among respondents aged 15 to 44 years (*n* = 4163), in whom accumulated chronic disease is uncommon (weighted mean of 0.89 conditions, multimorbidity of 22.3%), and disease-driven deterioration in economic circumstances or social contact has had less time to occur. Seventh, because reverse causation may be strongest for diagnosed mental health disorders, and because chronic pain is a heterogeneous condition, we recomputed the outcome without the mental health item, without the chronic-pain item, and without both, keeping the analytic sample, split, and model unchanged. Eighth, we repeated the analysis with a stricter non-response rule that treats only the codes labelled as “does not know” or “does not answer” in the questionnaire as missing. Finally, to test whether the neighbourhood items are simply proxies for material deprivation, we computed the weighted prevalence of the four leading neighbourhood problems by household income quintile and the largest absolute Spearman correlation between each of them and any variable in the socio-economic block.

### 2.8. Guide to the Analytical Steps

Table 1 summarises, for readers without a background in machine learning, what each step of the analysis does, why it was needed for this question, and how its output should be read.

## 3. Results

### 3.1. Analytic Sample and Outcome Distribution

Of the 16,590 respondents, 12,070 (72.8%) had complete data on the outcome and on the core demographic and behavioural indicators (Section 2.3), forming the analytic sample and representing approximately 12.36 million people aged 15 years or older. In this weighted sample, 50.0% were women, the mean age was 44.2 years, and 15.7% were aged 65 years or older, values consistent with the Chilean adult population. The mean weighted number of chronic conditions was 1.66, and the weighted multimorbidity prevalence was 40.1%. The condition count was right-skewed (unweighted skewness of +1.3), ranging from 0 to 19, with 27.7% of respondents reporting no conditions. Design-based analyses used 350 primary sampling units across 96 strata (Table 2).

### 3.2. Age Accounts for Almost a Quarter of the Variance

Design-weighted natural cubic splines of age explained 23.2% of the variance in condition count in the test set (Figure 2). This confirms age as the single dominant determinant and establishes the reference against which all subsequent contributions are measured: the residual outcome retained a weighted standard deviation of 1.69 conditions, and all reported second-stage quantities refer to this remaining variation.

### 3.3. Model Performance and the Value of Non-Linearity

**Figure 3:**
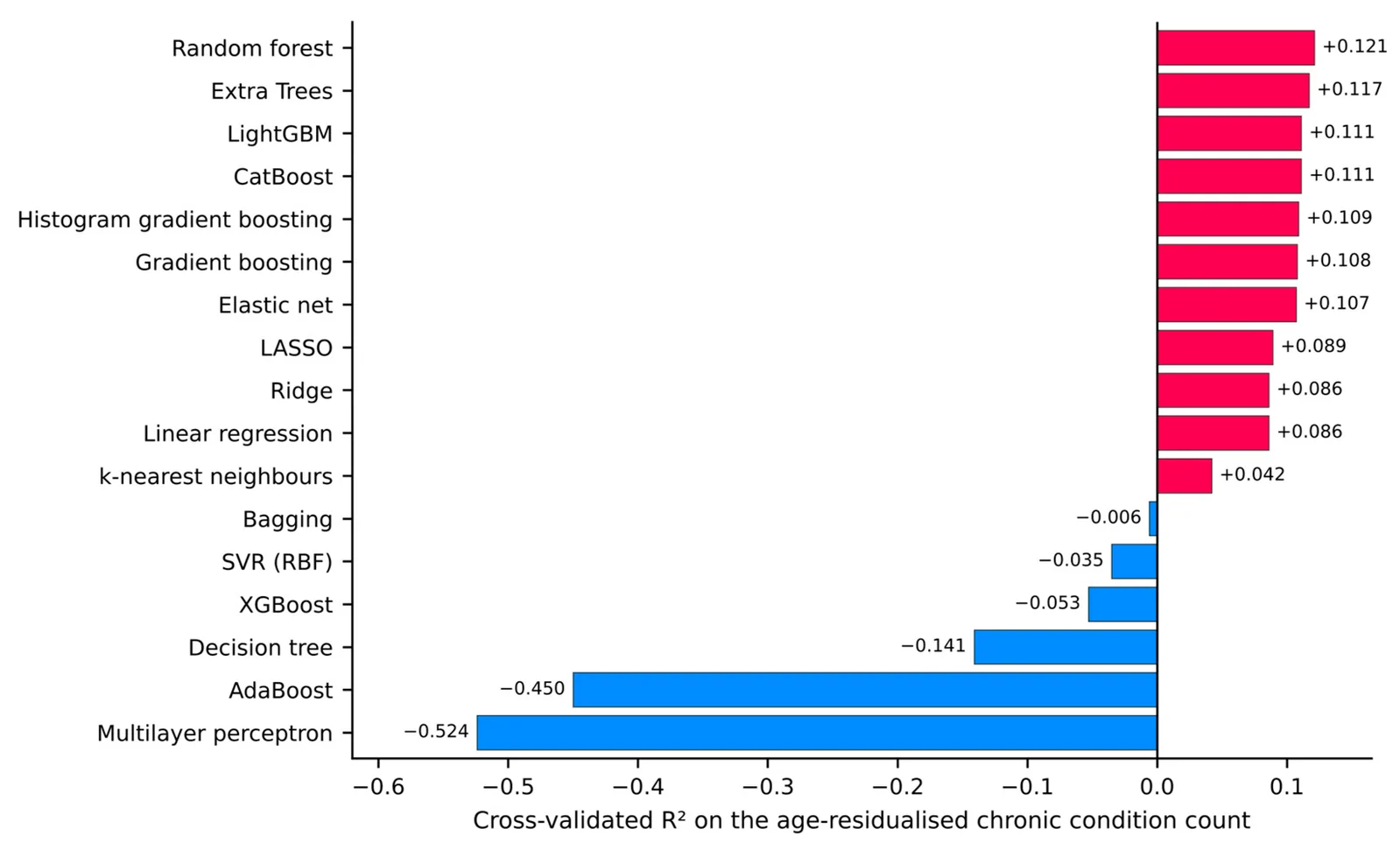
Cross-validated R^2^ of 17 supervised algorithms on the age-residualised chronic condition count. Tree ensembles (top) outperform linear models, although elastic net comes close. Red bars denote positive values and blue bars negative values, that is, prediction worse than the weighted mean (six algorithms).

Across the 17 algorithms, random forest achieved the highest cross-validated R^2^ on the age-residualised outcome (0.121), followed by Extra Trees (0.117), LightGBM (0.111), CatBoost (0.111), and histogram gradient boosting (0.109) (Table 3, Figure 3). Linear models performed worse, and not trivially so: elastic net reached 0.107, and ridge and unpenalised linear regression reached 0.086. Six algorithms produced negative R^2^ values, indicating worse out-of-fold prediction than the weighted mean: bagging, SVR, XGBoost, a single decision tree, AdaBoost, and the multilayer perceptron. With these fixed settings, random forest exceeded ridge regression by 0.035 and elastic net by 0.014 in cross-validated R^2^. Nested tuning narrowed the gap without closing it (Table S14): the tuned ridge, LASSO, and elastic net models reached a nested cross-validated R^2^ of 0.109 to 0.110, versus 0.125 for the tuned random forest and 0.132 for tuned LightGBM, the best model under tuning. A non-linear and interactive structure therefore adds approximately 0.015 to 0.022 in explained variance over a well-penalised linear model, which is a modest margin. On the held-out test set with disjoint primary sampling units, random forest achieved R^2^ = 0.117 (95% confidence interval (CI) of 0.091–0.138), MAE of 1.174 conditions (95% CI of 1.120–1.224), and an RMSE of 1.593 (95% CI of 1.512–1.675). Relative to a null model, these correspond to improvements of 5.3% in the MAE and 6.0% in the RMSE, and Cohen’s f^2^ was 0.132. Chaining the two stages, the full model explained 32.2% of the total variance in condition count. Put differently, roughly two-thirds of the variance in condition counts, and 88.3% of the variance remaining after age, was not explained by any of the determinants available in the survey; the attribution results that follow describe how the explained part is distributed and say nothing about the unexplained part.

### 3.4. Robustness

**Figure 4:**
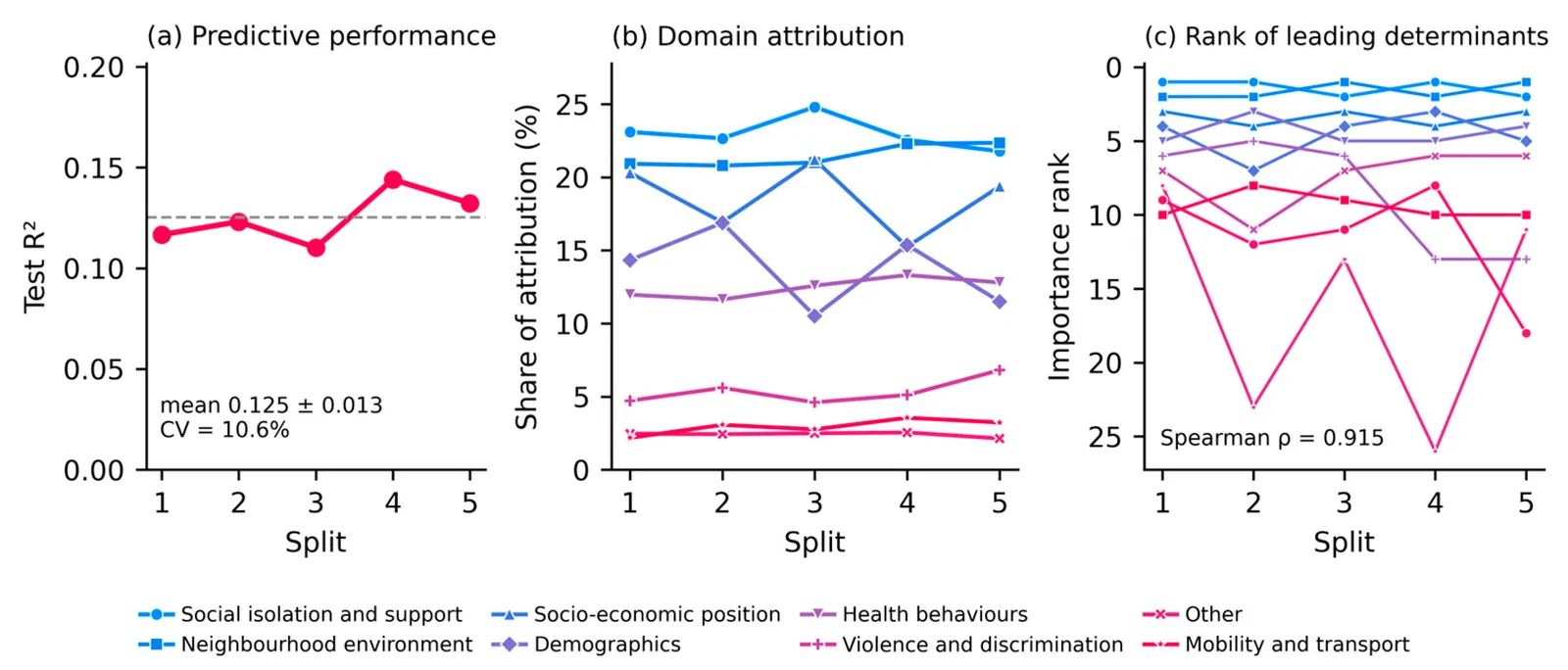
Robustness across five independent train–test splits grouped by primary sampling unit. (**a**) Test R^2^ by split; the dashed line marks the mean across splits, and the inset text gives the mean ± standard deviation and the coefficient of variation (CV). (**b**) Share of total attribution received by each domain in each split. (**c**) Importance rank of the ten leading determinants across splits; a flat line denotes a stable rank.

Performance was stable across five independent PSU-grouped splits: test R^2^ was 0.125 ± 0.013 (range from 0.110 to 0.144), the coefficient of variation was 10.6% of the mean, and the MAE varied between 1.137 and 1.224 conditions (Figure 4). The bootstrap confidence interval for test R^2^ excluded zero. The importance ranking was reproducible, with a mean pairwise Spearman correlation of 0.915 across seeds; six of the ten leading determinants appeared in the top ten of all five splits. Internal metric coherence was verified: the identity R^2^ = 1 − RMSE^2^/variance held to within 0.002 for all 17 algorithms, RMSE exceeded MAE in every case, and the ordering by R^2^ was identical to the ordering by RMSE. Domain attribution was recomputed on each split; each of the three leading social and environmental domains exceeded health behaviours in all five (Table S6).

The sensitivity analysis using principal components performed worse than the original variables (cross-validated R^2^ of 0.096 versus 0.121 for the same algorithm and folds) and, more importantly, precluded variable-level interpretation. Both are cross-validated values for the random forest and are therefore not directly comparable with the test R^2^ reported above. The determinant space showed no low-dimensional structure: the first component explained 5.6% of variance and 86 components were required to reach 80%. Dimensionality reduction was therefore not used in the primary analysis (Table S4).

### 3.5. Domain Attribution

**Figure 5:**
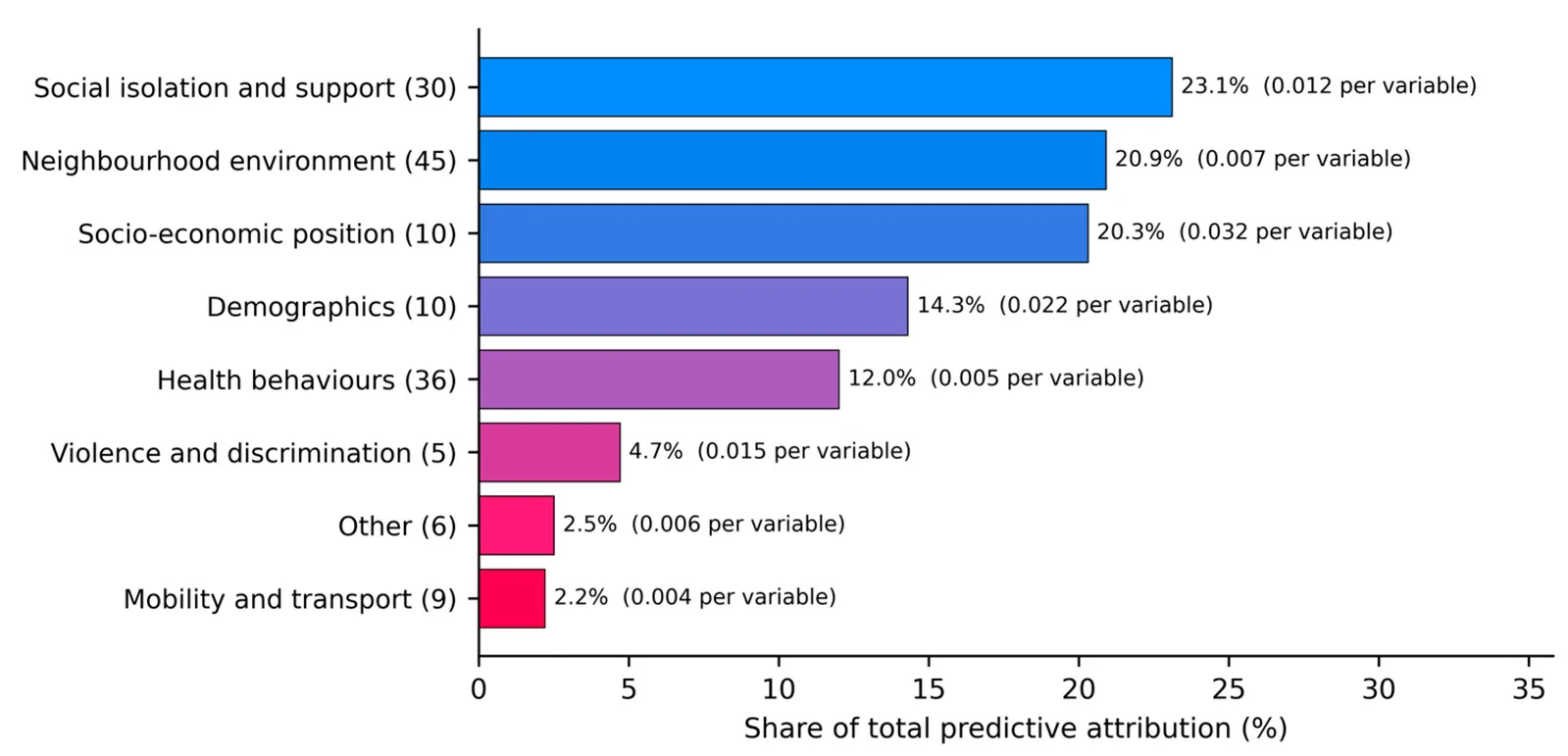
Domain-level attribution of the age-residualised chronic condition count, obtained by summing mean absolute SHAP values within pre-specified domains. Numbers in parentheses give the count of variables per domain; percentages give the share of total attribution, with the attribution per variable in parentheses. Bar colours are graded by rank for legibility and carry no additional information.

Aggregating SHAP attributions into pre-specified domains produced an ordering that inverts the conventional framing (Figure 5, Table 4). Social isolation and support received the most attribution (23.1% of the total across 30 variables), followed by neighbourhood environment (20.9%, 45 variables), socio-economic position (20.3%, 10 variables), and demographics (14.3%, 10 variables). Health behaviours, consisting of 36 variables covering physical activity, sedentary time, meals, alcohol, smoking, sleep, and screen use, received 12.0%. Violence and discrimination received 4.7% from only five variables.

Because these domains differ in size by a factor of nine, the same attribution is also reported per variable in Table 4 and under two further normalisations in Table S10. Normalising changes which domain leads, but under no normalisation do health behaviours exceed any of the three leading social and environmental domains. Per variable, socio-economic position received the most attribution (0.032), followed by demographics (0.022) and violence and discrimination (0.015), then social isolation and support (0.012) and neighbourhood environment (0.007); health behaviours received 0.005, which is less than any other substantive domain and one-sixth of the socio-economic figure. Restricting each domain to its five leading variables, so that every domain contributes an equal number of members, gives socio-economic position 24.6%, social isolation and support 20.2%, demographics 19.4%, neighbourhood environment 15.0%, health behaviours 7.7%, and violence and discrimination 6.7%. Therefore, normalising never lifts health behaviours: it ranks fifth out of eight on summed attribution, fifth out of eight under the five-variable restriction, and seventh out of eight per variable, below every other substantive domain. Its median member attribution (0.003) is indistinguishable from that of the neighbourhood environment (0.003), the only domain it reaches on that measure. The domains that lead do so with few strong members; the behavioural block is large and individually weak. Grouped attribution, which lets opposing contributions within a domain cancel, lowers health behaviours further (Table S13). In a re-run of the main specification, the grouped shares were 22.4% for socio-economic position, 21.1% for social isolation and support, 19.3% for demographics, and 16.7% for neighbourhood environment, versus 7.7% for health behaviours, which fell below violence and discrimination (8.3%). Permuting all members of a domain jointly painted the same picture: test R^2^ fell by 0.043 for socio-economic position, 0.040 for social isolation and support, and 0.025 for neighbourhood environment, but only by 0.011 for health behaviours. Averaging SHAP values with survey weights changed no domain share by more than 1.1 percentage points.

### 3.6. Leading Individual Determinants

**Figure 6:**
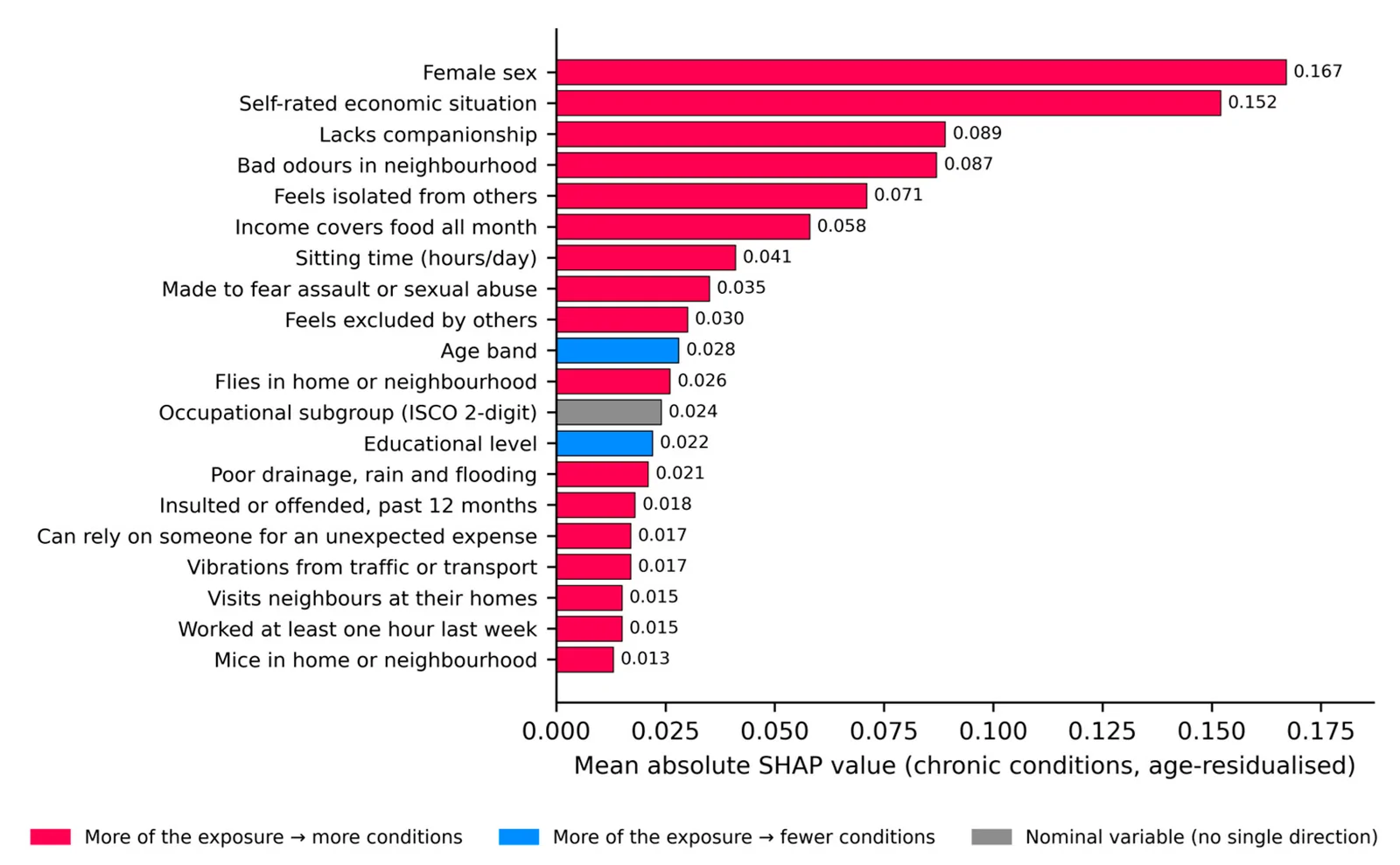
Twenty leading individual determinants by mean absolute SHAP value, with English descriptions of the survey items. The colour denotes the substantive direction of association: red indicates that more of the exposure (or the presence of the problem) is associated with a greater number of chronic conditions, blue is associated with fewer conditions, and grey is a nominal variable without a single direction.

At the variable level, the strongest contributors were female sex (mean |SHAP| of 0.167), poor self-rated economic situation of the respondent and their family (0.152), reporting a lack of companionship (0.089), bad odours in the neighbourhood (0.087), and feeling isolated from others (0.071) (Figure 6, Table 5). Income insufficient to cover food for the whole month ranked sixth (0.058), and daily sitting time, the first health behaviour variable to appear, ranked seventh (0.041). Having been made to fear assault or sexual abuse ranked eighth (0.035), and feeling excluded by others ranked ninth (0.030). Age band remained tenth (0.028) despite prior spline removal, indicating that a modest amount of age-related structure persists beyond a smooth function of chronological age. Its direction is inverse, with older bands associated with slightly fewer conditions than the spline predicts, which is consistent with a mild over-correction by the spline at older ages: in the test set, respondents aged 65 years or older had, on average, 0.25 fewer conditions than the first-stage curve predicted, whereas those aged 50 to 64 had 0.13 more (Figure 2); removing the age band left the domain ordering unchanged (Section 3.7). Educational level ranked thirteenth.

Below the top ten, the pattern remained consistent: the presence of flies in the home or neighbourhood, occupational subgroup, poor drainage and flooding, having been insulted or offended, the availability of someone to turn to for an unexpected expense, traffic vibrations, visiting neighbours at home, being in work last week, and the presence of mice. Smoking in the previous month appeared in the 43rd position. Once item polarity is taken into account, the directions were coherent throughout: greater material hardship, more isolation, more environmental deterioration, more exposure to violence and discrimination, and lower education were each associated with a higher condition count. This requires care because many ENCAVI items are coded 1 = yes and 2 = no, so that a higher raw value denotes absence of the problem; the Direction column of Table 5 is therefore expressed substantively, in terms of the exposure rather than the raw code, and the coding of every determinant is given in Table S2.

### 3.7. Sensitivity Analyses: Age, Sample Selection, Missing Data, and Younger Adults

Each social and environmental domain exceeded health behaviours regardless of how age was handled and with or without reweighting for the complete-case exclusion (Table 6; full domain shares in Table S8), although the order among the three leading domains changed when age was modelled directly. When age was not removed but modelled directly as a predictor, random forest explained 32.0% of the variance in the raw condition count on the test set, in line with the chained two-stage figure of 32.2%, and the two age variables together received 36.5% of the total attribution (continuous age: 24.0%, age band: 12.6%; shares rounded independently). Among the remaining determinants, socio-economic position received 27.8% of the attribution, social isolation and support received 20.1%, neighbourhood environment received 18.7%, health behaviours received 12.6%, demographics without age received 11.2%, and violence and discrimination received 3.9%. Each social and environmental domain therefore still exceeded health behaviours, and their combined share (66.6%) was, if anything, larger than in the main analysis. What changed was the order among the three leading domains: socio-economic position moved from third to first because several of its items track age closely; notably, having worked in the past 12 months, having worked last week, and marital status gained attribution once age was no longer removed. This is the redistribution of an age-related structure to age-related social variables that the two-stage design was intended to prevent.

Removing the age band from the residualised model left the ordering unchanged (social isolation and support: 23.8%, neighbourhood environment: 20.9%, socio-economic position: 20.0%, and health behaviours: 12.6%) and reduced test R^2^ only marginally (0.114 versus 0.117), so its retention in the main analysis did not distort the attribution of the other domains. Respondents excluded by the complete-case rule (*n* = 4520) differed modestly from those included: they were more often aged 65 years or older (19.1% versus 15.7%), more often women (54.4% versus 50.0%), and less often tertiary educated (32.4% versus 39.9%), and they reported material hardship slightly more often, whereas their multimorbidity prevalence, where the outcome was observed, was almost identical (40.3% versus 40.1%; Table S7). Two-thirds of the exclusions were due to a missing weekday sleep duration item, and 40% were due to a missing weight or height. Inclusion was patterned mainly by age and education in the weighted logistic model, with fitted inclusion probabilities between 0.46 and 0.87 among those included. Reweighting the analytic sample by the inverse of these probabilities left the domain shares within one percentage point of the main analysis (social isolation and support: 23.3%, neighbourhood environment: 20.9%, socio-economic position: 19.7%, and health behaviours: 12.1%) and test R^2^ essentially unchanged (0.118 versus 0.117).

Median imputation did not drive the ranking. Only 1.6% of the cells of the analytic matrix were imputed. Adding a missingness indicator for each of the 13 determinants with more than 5% missing values, so that the model could use the fact that a value had been imputed, left the domain shares within one percentage point of the main analysis (social isolation and support: 23.3%, neighbourhood environment: 20.7%, socio-economic position: 19.9%, and health behaviours: 12.2%) and test R^2^ at 0.115 versus 0.117. Dropping those 13 determinants instead of imputing them left the ordering unchanged but lowered health behaviours to 7.6%, because the block loses its two leading members, daily sitting hours and minutes; that figure is therefore a lower bound for the behavioural domain rather than an unbiased estimate of it, and the indicator analysis is the informative one. Details of the missing-data profile and of both refits are given in Table S11.

Three further analyses that modified the outcome count were run on the same split, and Table 6 compares them with a re-run of the main specification, whose domain shares differ from Table 4 by 0.5 percentage points at most. Removing mental health disorders from the count lowered the share of social isolation and support from 23.6% to 19.4%, and the attribution of lacking companionship fell by more than half (from 0.093 to 0.041), consistent with an overlap between loneliness and diagnosed depression or anxiety that may run in either direction. Each of the three social and environmental domains nonetheless still exceeded health behaviours (19.4%, 23.2%, and 21.7% versus 12.6%), and the same held when chronic pain or both items were removed. Repeating the attribution with other models preserved the contrast: with tuned LightGBM, the best model under tuning, health behaviours received 16.8% versus 17.7% to 22.0% for the three leading domains, and with tuned random forest and tuned elastic net, it received 13.1% and 9.3%. Finally, the stricter non-response rule changed no domain share by more than 0.5 percentage points, raising the test R^2^ from 0.117 to 0.120.

The one specification in which the ordering did not hold was the youngest part of the sample. Among the 4163 respondents aged 15 to 44 years, who reported, on average, 0.89 conditions and had a multimorbidity prevalence of 22.3%, health behaviours received the largest summed attribution (20.9%), ahead of social isolation and support (20.1%), neighbourhood environment (17.4%), violence and discrimination (14.4%), and socio-economic position (12.3%), with a test R^2^ of 0.101. Normalised per variable, violence and discrimination received by far the most attribution (0.027), followed by socio-economic position (0.011), demographics (0.008), and social isolation and support (0.006), with health behaviours ranking fifth out of eight (0.005). The subsample is about 2.9 times smaller than the analytic sample, and its outcome is much less variable, which limits the precision of these shares; their interpretation is taken up in Section 4.

## 4. Discussion

In a national Chilean survey, once age had been removed with flexible splines and downstream consequences of disease had been excluded by an explicit rule, social isolation, neighbourhood conditions, and material deprivation together received 64.3% of the predictive attribution for chronic condition burden, while health behaviours received 12.0%. This ordering is the central finding, and it runs against the framing that organises most chronic disease prevention policy. It is a statement about how a predictive model distributes its predictions among the variables available to it, not about causal contributions, and it applies only to the variation that the model captured: the majority of variability in multimorbidity remains unexplained, both before and after removing age. In absolute terms, these shares divide a model that explains 11.7% of the variance remaining after age; they are proportions of that explained part, not of the total burden of multimorbidity.

Four features give the result more weight than a single ranking would. First, after nested tuning of both families, the best tree ensemble still exceeded the best penalised linear model by about 0.02 in cross-validated R^2^, so part of the structure being detected is non-linear and interactive rather than an artefact of a particular functional form; the margin is real but moderate, and a well-specified linear model recovers most of the same signal. Second, the ranking was reproducible (Spearman ρ = 0.915) across independent partitions, which matters because impurity-based random-forest importance is unstable and frequently misused in medical applications [29], which is why SHAP was adopted as the primary measure [28]. Third, attribution was concentrated in few variables for socio-economic position and for violence and discrimination, and spread across many variables for neighbourhood environment, suggesting both concentrated and diffuse pathways rather than a single dominant proxy; and the position of health behaviours does not depend on domain size, since the behavioural block received the lowest attribution per variable of any substantive domain and did not rise when every domain was restricted to its five leading members. Fourth, the sensitivity analyses showed that each social and environmental domain exceeded health behaviours whether age was removed first or modelled directly, whether or not the age band was retained, whether or not missingness indicators were added, and whether or not the analytic sample was reweighted for exclusion; the ordering among the three leading domains, however, did depend on the handling of age, and the ordering as a whole reversed among adults aged under 45, a finding discussed in its own right below.

The prominence of social isolation is consistent with the strongest available evidence in the literature. A synthesis of 90 cohort studies links social isolation and loneliness to all-cause mortality [15], a finding replicated in a separate meta-analysis of loneliness [31], and isolation has been associated specifically with incident coronary heart disease and stroke [32]. Social support appears to mediate part of the pathway between social frailty and psychological distress [33], and the period of enforced physical distancing during the COVID-19 pandemic offered an unusually direct demonstration of the health cost of removing social contact [34]. Our finding that lacking companionship ranked third overall, with three isolation items among the top ten, places this domain at a magnitude comparable to material deprivation rather than subordinate to it. Longitudinal work linking cardiometabolic multimorbidity to social activity and joint trajectories of decline points to the same relationship over time [35].

The material findings that self-rated economic situation ranked second and income insufficient for food ranked sixth align with evidence that deprivation brings forward the onset of multimorbidity by a decade or more [12] and raises short-term mortality once chronic disease is established [13], and that disadvantage accumulates across the life course: intersecting trajectories beginning in childhood shape adult multimorbidity [36], and quasi-experimental evidence shows that exposures in the first 1000 days of life protect against chronic disease decades later [37]. Educational attainment and educational context operate as structural determinants in their own right [38], and social determinants shape disparities in hypertension control [39], the staging of cardiovascular–kidney–metabolic syndrome [40], and accelerated biological ageing [41], the latter offering one plausible mechanism through which these exposures may influence overall condition counts rather than individual diseases. When age was modelled directly rather than removed first, socio-economic position moved from third to first among the domains, because work-status and marital-status items that track age gained attribution; this is the redistribution that the two-stage design was intended to prevent, and it is why the main analysis reports contributions over and above age.

The neighbourhood findings are more unusual in their specificity. Bad odours ranked fourth, and flies, poor drainage, and traffic vibrations all entered the top twenty; the domain as a whole received more attribution than all health behaviours combined (20.9% versus 12.0%). These are markers of environmental deterioration and inadequate municipal services rather than of individual choice, and their direction agrees with evidence that place shapes multimorbidity [16], that poor housing and air pollution harm respiratory health [42,43], and that disadvantages embedded in place leave traces decades later [44]. Neighbourhood cohesion appears protective in the same literature [45,46]; here, visiting neighbours and neighbourhood companionship both entered the top twenty-five, with fewer conditions among the more connected.

A natural objection is that these items are proxies for material deprivation. They are patterned by it: bad odours were reported by 34% of respondents in the lowest household income quintile versus 17% in the highest, with shallower gradients of the same sign for the other three items (Table S12). They are not reducible to it, since the largest absolute correlation between any of the four and any socio-economic variable in the survey is 0.09. Even so, these indicators should be regarded as proxy markers rather than direct measures of environmental exposure: a reported odour, insect presence, or vibration has no dose and no objective referent, and may capture contextual deprivation, accumulated disadvantage, municipal service quality, and respondents’ perception of their surroundings as much as any physical agent. A predictive model cannot separate these components, and we make no claim that remediating odours would by itself alter chronic disease burden.

The contribution of violence and discrimination deserves particular comment because five variables received 4.7% of total attribution, an attribution per variable exceeded only by the socio-economic and demographic domains. Fear of assault ranked eighth overall, having been insulted ranked fifteenth, and experience of discrimination ranked twenty-seventh. This is compatible with evidence that escalating violence and associated fear of crime worsen psychological wellbeing [17]. It is also encouraging because the exposure is modifiable at the municipal scale: a citywide cluster randomised trial of blighted vacant-land remediation reduced gun violence, crime, and fear of crime [47]. These findings suggest that safety and dignity operate as health exposures in their own right rather than as contextual background.

Female sex was the single strongest determinant, and this finding needs careful reading. Women report more chronic conditions than men in most population surveys and primary-care records, a difference concentrated in mental, musculoskeletal, and pain-related conditions [1,48,49]. The same pattern holds here: women reported physician-diagnosed mental health disorders (25% versus 13%), joint disease (21% versus 8%), and chronic pain (20% versus 12%) far more often than men, and the attribution of female sex fell from 0.160 in the re-run of the main specification (Table 6, note d; 0.167 in Table 5) to 0.111 when mental health disorders were removed from the count and to 0.073 when chronic pain was also removed. Part of this difference is likely to reflect detection rather than occurrence, because women use health services more often and are therefore more likely to receive a diagnosis. Part of it is plausibly social: women were more likely than men to report that income did not cover food for the whole month (17% versus 11%) and to have been made to fear assault (12% versus 8%), and they carry a disproportionate share of unpaid care work, pathways that the literature on gender and health identifies as routes from social position to morbidity [50]. The finding therefore indicates that the social exposures examined here are unequally distributed by sex, and that diagnosed counts partly reflect access to diagnosis, rather than that sex itself is a modifiable determinant.

Health behaviours were not absent, as sitting time was the seventh strongest individual determinant, smoking appeared in the top fifty, and shared mealtimes contributed, but their aggregate attribution was smaller than that of three social and environmental domains, and their attribution per variable was smaller than that of every other substantive domain. This does not contradict the extensive evidence linking sedentary behaviour and exercise to cardiovascular health [10,51], the mechanistic literature on obesity and cardiovascular disease [52], or the associations of lifestyle factors with longitudinal progression and incident multimorbidity [11,53]. Two interpretations are possible and cannot be distinguished here. Behaviours may genuinely contribute less to condition accumulation than the social context in which they occur, an interpretation consistent with viewing behaviours as mediators of upstream exposures. Alternatively, self-reported behavioural measures may carry more measurement error than reports of neighbourhood conditions or economic hardship, attenuating their apparent contribution. We regard the second explanation as a genuine limitation rather than a rhetorical caveat, and note that it cannot be resolved with these data.

An attribution confined to condition counts understates the functional consequences of these exposures: multimorbidity clusters in reproducible patterns [54], is associated with frailty from middle age onward [55], and drives the loss of intrinsic capacity that predicts later outcomes [9], while disease management models remain built around single conditions [7], a mismatch that makes upstream determinants worth quantifying.

Reverse causation deserves more attention than a caveat, because it is the most plausible alternative explanation for the leading social findings. Chronic disease reduces earnings and employment, restricts participation in social life, and can change how people perceive and describe their surroundings. Self-rated economic situation, inability to cover food, lacking companionship, and feeling isolated—four of the eight leading determinants—are all open to this reading: a person with several conditions may report hardship and isolation because of the illness rather than before it. Three considerations bound the problem without solving it. First, the exclusion rule removed the variables most directly downstream of disease, and the cost of that choice is visible: retaining them raised the test R^2^ from 0.117 to 0.259, so roughly half of the apparent performance of an unrestricted model would have been the shadow of the disease. Second, the leading neighbourhood items describe conditions of the street rather than of the person, and it is harder to argue that an individual’s chronic conditions produce odours, flies, or poor drainage, although illness may increase the time spent at home and thus both exposure and attention to them. Third, removing diagnosed mental health disorders from the count, the item most exposed to this concern, lowered the share of social isolation and support by about four percentage points and halved the attribution of lacking companionship, although each social and environmental domain still exceeded health behaviours. The analysis of younger adults, discussed next, points the same way. Taken together, these considerations suggest that reverse causation inflates the social domains to an unknown degree and that the estimates for socio-economic position, in particular, should be read as upper bounds; only longitudinal data with exposures measured before disease onset can settle the question.

The result that most qualifies the main narrative comes from the youngest part of the sample, and we regard it as a finding in its own right rather than a failed robustness check. Among adults aged 15 to 44 years, health behaviours received the largest summed attribution (20.9%), ahead of social isolation and support (20.1%) and neighbourhood environment (17.4%); socio-economic position fell from third to fifth (12.3%), and violence and discrimination received by far the most attribution per variable. Two readings are compatible with this pattern and cannot be separated here. Under the first, material hardship and social isolation accumulate attribution in the full sample partly because chronic disease has already eroded income, employment, and social contact, an effect that has had little time to operate before 45; the fall in socio-economic position, with social isolation remaining second, is the pattern expected if part, but not all, of the material attribution reflects consequences of disease. Under the second, the determinants of early chronic disease differ from those of disease accumulated over a lifetime, with behaviours and interpersonal violence weighing more when conditions first appear and social disadvantage, which takes time to translate into accumulated disease, dominating later. Both imply age-specific aetiological pathways, with a practical corollary: prevention may need to be age-differentiated, with behavioural measures central in early adulthood and structural measures gaining weight thereafter, a proposition testable in cohorts that measure exposures before onset. The subsample is about 2.9 times smaller than the analytic sample and its outcome less variable, so this result is less precise than the main analysis and should be confirmed rather than assumed.

Several limitations require emphasis. The majority of variability in multimorbidity remains unexplained. Age accounted for 23.2% of the variance in condition counts, and the 151 determinants accounted for a further 9.0%; a total of 67.8% of the variance, including 88.3% of the variance remaining after age, was not captured by any variable in the survey. The percentages reported for each domain describe how the explained part is distributed and say nothing about the unexplained part, which may be distributed differently. The design is cross-sectional, and the consequences of that for reverse causation have been set out above. Effect sizes were small in absolute terms: the model improved prediction over a null model by 5.3% in mean absolute error, and Cohen’s f^2^ fell within the small range. These quantities describe relative attribution, not clinical prediction, and the model should not be used for individual risk estimation; established risk equations illustrate the standard that clinical prediction must meet [26,27]. The two-stage handling of age answers a question conditional on age; when age was modelled directly, the order among the three leading domains changed, and the age band retained a small residual contribution in the main analysis, so a modest amount of age-related structure remains shared among age-related social exposures. The outcome relies on self-reported physician diagnoses and is therefore sensitive to differential healthcare access, which may itself be patterned by the social exposures under study, including by sex. Complete-case analysis excluded 27.2% of respondents; reweighting by the inverse probability of inclusion did not change the domain shares (Section 3.7 and Table S7), but this corrects only selection on the variables in the inclusion model, and selection on unobserved characteristics, most plausibly related to the sleep item that accounted for most of the exclusions, cannot be ruled out. Finally, although the exclusion rule for downstream variables was explicit and conservative, no rule can guarantee that every retained variable is genuinely antecedent; self-rated economic situation, for instance, may be partly shaped by illness-related costs.

For Chile specifically, these results complement national evidence on multimorbidity in survey data, the association with tooth loss [21], and the mortality burden attributable to sitting time and physical inactivity [22], but place that burden alongside determinants that behavioural surveillance does not capture: one in three respondents reported bad odours in their neighbourhood, one in seven reported that their income did not cover food for the whole month, and one in ten reported having been made to fear assault. Chilean work has linked long-term adiposity to accelerated biological ageing in young adults [56], documented a rising chronic kidney disease burden [57], and described social gradients in nutritional biomarkers [58] and household food insecurity [59]; what has been missing is an estimate of the weight of these exposures relative to behaviours.

From a policy perspective, the findings do not argue against behavioural interventions, which have independent justification. They suggest that a prevention strategy addressing only behaviours would concentrate on the domain that received the least predictive attribution of those examined, whether attribution is summed or normalised for domain size, within a model that leaves most of the variation unexplained. Municipal environmental services, food security, social connection, and neighbourhood safety received a larger predictive attribution; this design cannot show that acting on them would reduce multimorbidity, but they are amenable to policy at scales where health systems alone cannot act.

## 5. Conclusions

After accounting for age, social isolation and support, neighbourhood environment, and socio-economic position together accounted for 64.3% of predictive attribution within the final model among Chilean adults, compared with 12.0% for health behaviours; the model explained 11.7% of the variance remaining after age. Each of the three domains remained above health behaviours across the analytical choices most likely to bias the comparison: how age is handled, how missing data and exclusions are treated, and how the domains are normalised for size. The contrast reversed among adults aged 15 to 44 years, in whom health behaviours received the largest attribution, which points to age-specific pathways into multimorbidity, possibly with behaviours weighing more when conditions first appear and social disadvantage, which takes time to translate into accumulated disease, weighing more later. These associations come from a cross-sectional predictive model: attribution is not causal attribution, individual effects are modest, most of the burden of multimorbidity remains unexplained, and reverse causation cannot be ruled out for the social domains. Within those limits, the findings question the assumption that multimorbidity prevention in the adult population as a whole should rest primarily on changing individual behaviour. Social isolation, neighbourhood environments, and socio-economic inequalities warrant further investigation as potential targets for prevention policies, and their effects, together with the life stages at which they operate, should be evaluated in longitudinal and intervention studies.

## Figures and Tables

**Table 1:** Plain-language guide to the analytical steps.

Step	What It Does	Why It Was Needed	How to Read Its Output
Survey weights, strata, and PSUs	Each respondent stands for a different number of Chileans; the weight restores that proportion, and strata and PSUs are the clusters from which respondents were sampled.	Unweighted estimates describe the sample rather than the population, and ignoring clusters makes results look more precise than they are.	All percentages, means, and performance metrics are population estimates.
Condition count as an outcome	Counts the 19 physician-diagnosed conditions each respondent reports.	A count keeps information that a yes/no multimorbidity label discards, including the upper tail.	Model errors are in units of conditions.
Exclusion of downstream variables	Removes variables that are consequences of disease, such as self-rated health or functional limitations.	Consequences predict disease well but explain nothing about its causes; keeping them inflates performance.	Performance is lower than in models that keep such variables by design.
Two-stage age adjustment	Fits a flexible curve of condition count on age and keeps the difference between each respondent’s count and that curve.	Age is the strongest determinant and is correlated with most social variables; removing it first prevents its effect from being credited to them.	All second-stage results describe variation among people of the same age.
Train/test split by PSU	Whole sampling clusters are assigned to either the training or the test set.	Respondents from the same cluster resemble each other; splitting them across sets would make the test optimistic.	Test metrics are estimates of performance in new clusters.
Cross-validation	Repeatedly fits each algorithm on four-fifths of the training data and scores it on the remaining one-fifth.	Chooses the algorithm without using the test set.	Cross-validated R^2^ compares algorithms; test R^2^ reports the chosen one.
Panel of 17 algorithms	Compares model families from linear regression to tree ensembles and neural networks, with the panel and the selection rule fixed in advance.	Tests, rather than assumes, whether a non-linear and interactive structure is present, and removes the choice of algorithm as a researcher’s degree of freedom.	A large gap between tree ensembles and linear models indicates a non-linear structure; a small gap means that a linear model would tell a similar story.
R^2^, MAE, and RMSE	R^2^ is the share of variance explained; MAE and RMSE are average prediction errors in conditions.	Reported against a null model that predicts the mean, so that improvement is explicit.	An R^2^ near zero means that little is explained; a negative R^2^ means that it is worse than the mean.
SHAP attribution	Splits each prediction among the variables that produced it and averages the absolute contributions.	Impurity-based importance in random forests favours continuous variables; SHAP does not.	Larger values mean a larger role in the model’s predictions, not a larger causal effect.
Domain shares	Sums attribution within groups of variables defined before modelling and expresses each as a percentage of the total.	The question concerns groups of exposures, not single items.	Percentages sum to 100 and describe only the variation the model captured.
Bootstrap and repeated splits	Recomputes metrics on 1000 resamples and repeats the whole pipeline on five splits.	Shows how much the results depend on the particular respondents or partition.	Narrow intervals and stable ranks indicate reproducible findings.
Sensitivity analyses	Repeats attribution without age residualisation, without age band, with inclusion weights, with missingness indicators, among adults under 45, without mental health or chronic pain in the count, with other models, and with principal components.	Tests whether the domain ordering depends on these analytical choices.	Whether health behaviours stay below each social and environmental domain is the relevant check.

PSU, primary sampling unit; MAE, mean absolute error; RMSE, root mean squared error; SHAP, SHapley Additive exPlanations.

**Table 2:** Weighted characteristics of the analytic sample, overall and by multimorbidity status.

Characteristic	All	No Multimorbidity (<2 Conditions)	Multimorbidity (≥2 Conditions)
Unweighted *n*	12,070	5867	6203
Weighted prevalence	100.0	59.9	40.1
Age, mean (years)	44.2	37.8	53.7
Aged 65 years or older	15.7	6.1	30.1
Women	50.0	44.4	58.4
Tertiary education	39.9	44.4	33.2
Self-rated poor economic situation	8.6	5.3	13.5
Income does not cover food for the month	13.2	10.4	17.3
Often lacks companionship	11.0	7.8	15.9
Often feels isolated from others	6.5	4.3	9.8
Bad odours in their neighbourhood	30.5	26.6	36.4
Made to fear assault or abuse	10.0	7.8	13.3
Experienced discrimination	7.0	5.7	8.9
Body mass index, mean (kg/m^2^)	28.0	27.3	29.0
Sitting time, mean (hours/day)	4.05	4.03	4.07
Moderate-to-vigorous physical activity, mean (min/week)	226	268	163
Sleep duration, mean (hours)	6.8	6.9	6.7
Chronic conditions, mean (*n*)	1.66	0.40	3.54

Values are weighted percentages unless the row label indicates a mean, in which case the unit is given in parentheses; only the unweighted *n* is not weighted. All values are weighted by the calibrated person weight. Multimorbidity is defined as two or more of 19 physician-diagnosed chronic conditions. Percentages for ordinal survey items refer to the most severe response category.

**Table 3:** Performance of 17 supervised algorithms on the age-residualised chronic condition count (five-fold cross-validation with PSU grouping, design-weighted), with the software module used for each.

Algorithm	R^2^	MAE	RMSE	Module (Library)
Random forest	+0.121	1.176	1.612	ensemble.RandomForestRegressor (scikit-learn)
Extra Trees	+0.117	1.176	1.616	ensemble.ExtraTreesRegressor (scikit-learn)
LightGBM	+0.111	1.170	1.621	LGBMRegressor (lightgbm)
CatBoost	+0.111	1.179	1.621	CatBoostRegressor (catboost)
Histogram gradient boosting	+0.109	1.173	1.622	ensemble.HistGradientBoostingRegressor (scikit-learn)
Gradient boosting	+0.108	1.169	1.624	ensemble.GradientBoostingRegressor (scikit-learn)
Elastic net	+0.107	1.178	1.624	linear_model.ElasticNet (scikit-learn)
LASSO	+0.089	1.192	1.641	linear_model.Lasso (scikit-learn)
Ridge	+0.086	1.204	1.643	linear_model.Ridge (scikit-learn)
Linear regression	+0.086	1.204	1.643	linear_model.LinearRegression (scikit-learn)
k-nearest neighbours	+0.042	1.175	1.682	neighbors.KNeighborsRegressor (scikit-learn)
Bagging	−0.006	1.271	1.725	ensemble.BaggingRegressor (scikit-learn)
SVR (RBF)	−0.035	1.296	1.749	svm.SVR (scikit-learn)
XGBoost	−0.053	1.301	1.765	XGBRegressor (xgboost)
Decision tree	−0.141	1.344	1.836	tree.DecisionTreeRegressor (scikit-learn)
AdaBoost	−0.450	1.747	2.070	ensemble.AdaBoostRegressor (scikit-learn)
Multilayer perceptron	−0.524	1.615	2.122	neural_network.MLPRegressor (scikit-learn)

Values are design-weighted, five-fold cross-validated means on the age-residualised outcome, with primary sampling units held together across folds. The null model predicting the weighted mean has R^2^ = 0 and an RMSE equal to the design-weighted standard deviation of the residual outcome in the training set used for cross-validation (1.719; the corresponding value in the test set is 1.69, Figure 2 and Section 3.2). Negative R^2^ denotes out-of-fold prediction worse than the mean. Module names are given relative to the library’s top-level package. SVR (RBF)—support vector regression with a radial basis function kernel. MAE, mean absolute error; RMSE, root mean squared error; PSU, primary sampling unit.

**Table 4:** Domain-level attribution of the age-residualised chronic condition count, summed and per variable.

Domain	Variables, *n*	Summed Mean |SHAP|	% of Total Attribution	Mean |SHAP| per Variable	Rank per Variable
Social isolation and support	30	0.360	23.1	0.012	4
Neighbourhood environment	45	0.326	20.9	0.007	5
Socio-economic position	10	0.316	20.3	0.032	1
Demographics	10	0.223	14.3	0.022	2
Health behaviours	36	0.186	12.0	0.005	7
Violence and discrimination	5	0.073	4.7	0.015	3
Other	6	0.038	2.5	0.006	6
Mobility and transport	9	0.034	2.2	0.004	8

Domains were pre-specified by variable prefix before attribution was computed. “Other” comprises household composition and area variables not assignable to a substantive domain. Percentages were computed from unrounded attribution totals and sum to 100 within rounding; recomputing them from the three-decimal values shown may differ by up to 0.1 percentage points. The last two columns normalise attribution for the number of variables a domain contains, because a domain with more members has more opportunity to accumulate attribution; two further normalisations are given in Table S10. SHAP, SHapley Additive exPlanations.

**Table 5:** Twenty leading individual determinants of the age-residualised chronic condition count.

#	Determinant	Domain	Mean |SHAP|	Direction (Item Coding)
1	Female sex	Demographics	0.167	Women → more (1 = man, 2 = woman)
2	Self-rated economic situation	Socio-economic	0.152	Worse situation → more (1 = good … 3 = poor)
3	Lacks companionship	Social isolation	0.089	More often → more (1 = almost never … 3 = often)
4	Bad odours in neighbourhood	Neighbourhood	0.087	Problem present → more (1 = yes, 2 = no)
5	Feels isolated from others	Social isolation	0.071	More often → more (1 = almost never … 3 = often)
6	Income covers food all month	Socio-economic	0.058	Does not cover → more (1 = yes, 2 = no)
7	Sitting time (hours/day)	Health behaviours	0.041	More sitting → more (hours per day)
8	Made to fear assault or sexual abuse	Violence	0.035	Exposure present → more (1 = yes, 2 = no)
9	Feels excluded by others	Social isolation	0.030	More often → more (1 = almost never … 3 = often)
10	Age band	Demographics	0.028	Older band → fewer (residual) (1 = 15–19 … 5 = 65 or older)
11	Flies in home or neighbourhood	Neighbourhood	0.026	Problem present → more (1 = yes, 2 = no)
12	Occupational subgroup (ISCO 2-digit)	Socio-economic	0.024	Nominal; see Table S2 (ISCO code)
13	Educational level	Socio-economic	0.022	Higher education → fewer (1 = primary … 4 = tertiary)
14	Poor drainage, rain, and flooding	Neighbourhood	0.021	Problem present → more (1 = yes, 2 = no)
15	Insulted or offended, past 12 months	Violence	0.018	Exposure present → more (1 = yes, 2 = no)
16	Can rely on someone for an unexpected expense	Social isolation	0.017	Less able to rely → more (1 = always … 4 = rarely/never)
17	Vibrations from traffic or transport	Neighbourhood	0.017	Problem present → more (1 = yes, 2 = no)
18	Visits neighbours at their homes	Social isolation	0.015	Less visiting → more (1 = strongly agree … 4 = disagree)
19	Worked at least one hour last week	Socio-economic	0.015	Not working → more (1 = yes, 2 = no)
20	Mice in home or neighbourhood	Neighbourhood	0.013	Problem present → more (1 = yes, 2 = no)

Attribution from TreeExplainer on the held-out test set. The direction states the substantive association: the effect of more of the exposure, with the raw item coding given in parentheses. Many ENCAVI items are coded 1 = yes and 2 = no, so a higher raw value denotes absence of the problem. Age band remains present because the spline stage removes only a smooth function of chronological age. Domain names are shortened: Social isolation, social isolation and support; Neighbourhood, neighbourhood environment; Socio-economic, socio-economic position; Violence, violence and discrimination (full names as in Table 4). ISCO, International Standard Classification of Occupations. Full wording and coding for all 151 determinants, with English descriptions, are in Table S2. SHAP, SHapley Additive exPlanations.

**Table 6:** Domain attribution under alternative specifications.

Analysis	Test R^2^	Social Isolation	Neighbourhood	Socio-Economic	Demographics	Health Behaviours	Violence
Main analysis (Table 4)	0.117	23.1	20.9	20.3	14.3	12.0	4.7
No residualisation, age as predictor: all variables	0.320 ^a^	12.8	11.8	17.6	43.7 ^b^	8.0	2.5
No residualisation, age as predictor: age variables excluded and shares renormalised	0.320 ^a^	20.1	18.7	27.8	11.2	12.6	3.9
Age-residualised, age band removed	0.114	23.8	20.9	20.0	12.5	12.6	5.2
Age-residualised, inclusion-probability weighted	0.118	23.3	20.9	19.7	14.6	12.1	5.0
Missingness indicators added for the 13 determinants above 5% missing	0.115	23.3	20.7	19.9	14.1	12.2	5.1
Those 13 determinants dropped instead of imputed	0.111	25.5	21.4	19.1	15.3	7.6 ^c^	5.6
Adults aged 15 to 44 years (*n* = 4163)	0.101	20.1	17.4	12.3	8.6	20.9	14.4
Re-run of the main specification ^d^	0.117	23.6	20.4	19.9	13.9	11.9	5.2
Mental health disorders removed from the count ^d^	0.100	19.4	23.2	21.7	12.5	12.6	5.1
Chronic pain removed from the count ^d^	0.108	23.4	20.3	21.6	12.9	12.2	4.7
Both items removed from the count ^d^	0.091	19.1	23.7	23.4	10.8	12.5	4.3
Tuned LightGBM, best model under nested tuning ^d^	0.135	21.0	22.0	17.7	10.3	16.8	4.6
Tuned random forest ^d^	0.122	23.8	22.2	18.2	11.9	13.1	5.3
Tuned elastic net, linear attribution ^d^	0.113	22.6	20.5	18.9	10.3	9.3	6.3
Stricter non-response rule ^d^	0.120	23.2	20.6	19.5	14.3	12.4	5.3

Values are the percentage of total mean absolute SHAP attribution received by each domain on the held-out test set of the seed-42 split; the “other” and “mobility and transport” domains, which together received the remainder (between 3.6% and 12.1%, depending on the specification), are given in Table S8. ^a^ R^2^ for the raw condition count rather than for the age-residualised outcome, so it is comparable with the chained figure of 32.2% and not with the other rows. ^b^ Includes continuous age (24.0%) and the age band (12.6%). ^c^ The behavioural block loses its two leading members, daily sitting hours and minutes, when those determinants are dropped, so 7.6% is a lower bound for that domain rather than an unbiased estimate. Except for the tuned LightGBM, tuned random forest, and tuned elastic net rows, all rows were produced with the estimator of record (random forest, 300 trees, minimum leaf size 20, seed 42) on the same split, so the first row is Table 4 itself, and the other rows that use this estimator are directly comparable with it. ^d^ Additional analyses run on the same split and compared with the re-run of the main specification in the same block, whose shares differ from Table 4 by 0.5 percentage points at most; the tuned models use the settings selected in Table S14, and the elastic net attribution is the coefficient multiplied by the centred value. Column headings give shortened domain names: Social Isolation, social isolation and support; Neighbourhood, neighbourhood environment; Socio-Economic, socio-economic position; Violence, violence and discrimination (full names as in Table 4). SHAP, SHapley Additive exPlanations.

## Data Availability

The ENCAVI 2023–2024 dataset is publicly available through the Chilean Ministry of Health. The analysis code (random seed 42) reproducing every figure, table, and reported value is deposited in GitHub at https://github.com/datasciencex1x-ux/Environmental-Determinants- (the repository name ends with a hyphen; accessed on 1 October 2026).

## Supplementary Materials

The following supporting information can be downloaded at: https://www.mdpi.com/article/10.3390/healthcare14213565/s1, Table S1: Variable exclusion log; Table S2: Complete SHAP attribution for all 151 determinants; Table S3: Per-split robustness; Table S4: Principal-component sensitivity analysis; Table S5: Domain aggregation; Table S6: Stability of domain attribution across splits; Table S7: Characteristics of respondents included in and excluded from the analytic sample; Table S8: Domain attribution in the sensitivity analyses; Table S9: Design-weighted logistic model of inclusion in the analytic sample; Table S10: Domain attribution under four normalisations; Table S11: Missing-data profile and sensitivity; Table S12: Leading neighbourhood problems by household income quintile; Table S13: Domain attribution under grouped and survey-weighted measures; Table S14: Nested hyperparameter tuning of the penalised linear models and tree ensembles.
