# Supplementary material for: Social and Environmental Determinants Show Greater Predictive Attribution than Health Behaviours for Multimorbidity: A Population-Based Machine Learning Study
Source: Healthcare (Basel). 2026 Oct 22;14(21):3565. doi: 10.3390/healthcare14213565 (PMC13762737; doi:10.3390/healthcare14213565)
Supplement: Supplementary file 1 [file healthcare-14-03565-s001.zip › healthcare-4523057-supplementary.pdf]

Supplementary Materials

Social and Environmental Determinants Show Greater Predictive Attribution Than Health Behaviours for Multimorbidity: A Population-Based Machine Learning Study

All values were produced by the deposited analysis code under random seed 42 on the 2023–2024 ENCAVI public-use file (16,590 respondents, 464 variables). Abbreviations used in these tables: SHAP, SHapley Additive exPlanations; PSU, primary sampling unit; MAE, mean absolute error; RMSE, root mean squared error; PCA, principal component analysis; ISCO, International Standard Classification of Occupations; ISIC, International Standard Industrial Classification; IPAQ, International Physical Activity Questionnaire.

Table S1. Variable exclusion log

Sequence applied to reduce the 464 available variables to 151 candidate upstream determinants. Every step was specified before any model was fitted; the same sequence is shown as Figure 1 of the main text. Step 3 counts only variables not already removed in steps 1–2; the submitted version counted 20 outcome-derived items twice and did not list the free-text fields (step 5b).

Step	Variables removed	Reason
1–2. Outcome and outcome-derived	41	Define or are derived from the outcome
3. Functional capacity module	45	Consequence of chronic disease
4. Health-status proxies and service use	24	Downstream of disease / reverse causation
5. Design and administrative	9	Design and administrative
5b. Free-text and “other, specify” fields	10	Not analysable as determinants
6. Missingness / cardinality / zero variance	135	Data quality
6b. WHOQOL-BREF composite scores	3	Derived and downstream
7. Near-duplicates (|r| > 0.95)	7	Redundant information
8. Subjective wellbeing and functioning items	37	Measured concurrently, downstream
9. Body-image items	2	Reflect adiposity and illness-related distress

The rule is deliberately conservative: any variable plausibly measuring a consequence of chronic disease was excluded, even at the cost of discarding informative predictors. Retaining them raised test R^2 from 0.117 to 0.259 in a preliminary specification, illustrating how much apparent performance reverse causation can generate; that specification is not reported as a result.

Table S2. Complete SHAP attribution for all 151 determinants

Mean absolute SHAP values from TreeExplainer applied to the random forest on the held-out test set. The English description column gives the meaning of each survey item; the survey wording is kept in Spanish so that the item can be located in the ENCAVI questionnaire. The coding column gives the polarity of each survey item, which is required to read the direction of association: many ENCAVI items are coded 1 = yes and 2 = no, so a higher raw value denotes absence of the problem.

Rank	Code	Survey item (Spanish wording)	English description	Item coding	Mean |SHAP|	% of total
1	sexo	Sexo del seleccionado/a	Sex of respondent	1 = Hombre; 2 = Mujer	0.16749	10.754
2	p2_3	¿Cómo calificaría en general su situación económica actual y la de su familia?	Self-rated economic situation of respondent and family	1 = Buena; 2 = Ni buena ni mala; 3 = Mala ...	0.15228	9.777
3	p8_5_1	(Siente que le falta compañía) Las siguientes preguntas son acerca de cómo se siente Ud. en diferentes aspecto	Feels lacking companionship (frequency)	1 = Casi nunca; 2 = A veces; 3 = A menudo ...	0.08924	5.730
4	p8_11_5	(Malos olores) ¿Qué problemas relacionados de contaminación o deterioro del entorno identifica usted en su bar	Bad odours identified as a neighbourhood problem	1 = Sí; 2 = No; 8 = No sabe (NO LEER) ...	0.08746	5.616
5	p8_5_3	(Se siente aislado/a de los demás) Las siguientes preguntas son acerca de cómo se siente Ud. en diferentes asp	Feels isolated from others (frequency)	1 = Casi nunca; 2 = A veces; 3 = A menudo ...	0.07103	4.560
6	p11_17_2	(¿Los alimentos para comer durante todo el mes?) Y, diría que el ingreso mensual del hogar les permite pagar s	Household income covers food for the whole month	1 = Sí; 2 = No; 8 = No sabe (NO LEER) ...	0.05753	3.694
7	p5_10_1_t	Tiempo sentado: Horas por día	Sitting time, hours per day	continuous	0.04051	2.601
8	p9_1_4	(Hizo que sintiera miedo de sufrir un ataque, asalto o abuso sexual en la calle) Durante los últimos 12 meses,	Someone made respondent fear attack, assault or sexual abuse in the street, past 12 months	1 = Sí; 2 = No; 88 = No sabe (NO LEER) ...	0.03459	2.221
9	p8_5_2	(Se siente excluido/a o dejado/a de lado por los demás) Las siguientes preguntas son acerca de cómo se siente	Feels excluded or left aside by others (frequency)	1 = Casi nunca; 2 = A veces; 3 = A menudo ...	0.02952	1.895

10	edad5	Tramos etarios	Age band	1 = 15 a 19 años; 2 = 20 a 29 años; 3 = 30 a 49 años ...	0.02796	1.795
11	p8_12_1	(Moscas) En su casa o barrio, ¿ha notado la presencia de…?	Flies noticed in home or neighbourhood	1 = Sí; 2 = No; 8 = No sabe (NO LEER) ...	0.02582	1.658
12	SUBGRUPO_CIUO	Clasificación Internacional Uniforme de Ocupaciones: Subgrupos grupos (2 dígitos)	Occupational subgroup (ISCO, two digits)	-1 = No sabe o No responde; 2 = Suboficiales; 3 = Otros miembros ...	0.02351	1.510
13	educ	Nivel educacional (4 grupos)	Educational level (four groups)	1 = Básica incompleta o completa; 2 = Media incompleta; 3 = Media completa ...	0.02187	1.404
14	p8_11_7	(Deficiente evacuación de aguas – lluvias e inundaciones) ¿Qué problemas relacionados de contaminación o deter	Poor drainage, rain and flooding identified as a neighbourhood problem	1 = Sí; 2 = No; 8 = No sabe (NO LEER) ...	0.02141	1.374
15	p9_1_1	(Le ha insultado u ofendido) Durante los últimos 12 meses, le ha pasado que una persona conocida o desconocida	Insulted or offended by someone, past 12 months	1 = Sí; 2 = No; 88 = No sabe (NO LEER) ...	0.01828	1.174
16	p8_4	¿Puede recurrir confiadamente a alguien cuando tiene un gasto imprevisto, emergencia económica u otra situació	Can confidently turn to someone for an unexpected expense or economic emergency	1 = Sí, siempre; 2 = Sí, casi siempre; 3 = Sí, algunas veces ...	0.01716	1.102
17	p8_11_10	(Vibraciones (autopistas, metro, paso de camiones)) ¿Qué problemas relacionados de contaminación o deterioro d	Vibrations (motorways, metro, lorries) identified as a neighbourhood problem	1 = Sí; 2 = No; 8 = No sabe (NO LEER) ...	0.01671	1.073
18	p8_9_3	(Visito a mis vecinos en sus casas) ¿Cuán de acuerdo está con las siguientes frases?	Agreement: I visit my neighbours in their homes	1 = Muy de acuerdo; 2 = De acuerdo; 3 = Ni de acuerdo ni en desacuerdo ...	0.01515	0.973
19	p7_2	La semana pasada, ¿trabajó al menos una hora, sin considerar los quehaceres del hogar?	Worked at least one hour last week (excluding housework)	1 = Sí; 2 = No; 8 = No sabe (NO LEER) ...	0.01481	0.951
20	p8_12_3	(Ratones) En su casa o barrio, ¿ha notado la presencia de…?	Mice noticed in home or neighbourhood	1 = Sí; 2 = No; 8 = No sabe (NO LEER) ...	0.01335	0.857
21	p8_12_6	(Garrapatas) En su casa o barrio, ¿ha notado la presencia de…?	Ticks noticed in home or neighbourhood	1 = Sí; 2 = No; 8 = No sabe (NO LEER) ...	0.01325	0.851
22	p8_9_16	(Tengo un sentimiento de compañerismo profundo con otras personas de este barrio) ¿Cuán de acuerdo está con la	Agreement: I have a deep feeling of companionship with other people in this neighbourhood	1 = Muy de acuerdo; 2 = De acuerdo; 3 = Ni de acuerdo ni en desacuerdo ...	0.01321	0.848
23	p8_11_6	(Microbasurales) ¿Qué problemas relacionados de contaminación o deterioro del entorno identifica usted en su b	Illegal dumping sites identified as a neighbourhood problem	1 = Sí; 2 = No; 8 = No sabe (NO LEER) ...	0.01247	0.801
24	p2_13_1_b	(Usar redes sociales como Instagram, Facebook, TikTok o Twitter) Semana	Weekday hours using social media	continuous	0.01241	0.797
25	p5_1_1_b	(Desayuno) ¿Y con quién comparte esta comida la mayoría de las veces?	With whom breakfast is usually shared	1 = Solo; 2 = Con familiares; 3 = Con amigos ...	0.01233	0.792
26	p8_12_2	(Zancudos o mosquitos) En su casa o barrio, ¿ha notado la presencia de…?	Mosquitoes noticed in home or neighbourhood	1 = Sí; 2 = No; 8 = No sabe (NO LEER) ...	0.01224	0.786
27	p8_14	En los últimos 12 meses, ¿usted se ha sentido discriminado?	Felt discriminated against, past 12 months	1 = Sí; 2 = No; 8 = No sabe (NO LEER) ...	0.01189	0.763
28	p6_1_4_a	(Automóvil) En los últimos 30 días, ¿ha conducido uno de los siguientes vehículos: automóvil, motocicleta y bi	Drove a car in the past 30 days	1 = Sí; 2 = No	0.01186	0.761
29	p8_11_8	(Perros vagos) ¿Qué problemas relacionados de contaminación o deterioro del entorno identifica usted en su bar	Stray dogs identified as a neighbourhood problem	1 = Sí; 2 = No; 8 = No sabe (NO LEER) ...	0.01180	0.758
30	p1_15	Último curso aprobado	Highest school year completed	continuous	0.01178	0.757
31	trabajador	Trabajador últimos 12 meses	Worked in the past 12 months	1 = Trabajado/ar; 2 = No trabajador	0.01034	0.664
32	sum_org_cat	Suma de participación en organizaciones formales categorizada	Number of formal organisations in which respondent participates (categorised)	1 = Cero; 2 = Una; 3 = Dos o más	0.00982	0.631
33	p8_9_14	(Me gusta sentirme parecido a las personas que viven en mi barrio) ¿Cuán de acuerdo está con las siguientes fr	Agreement: I like feeling similar to the people who live in my neighbourhood	1 = Muy de acuerdo; 2 = De acuerdo; 3 = Ni de acuerdo ni en desacuerdo ...	0.00972	0.624
34	p5_10_2_t	Tiempo sentado: Minutos por día	Sitting time, additional minutes per day	continuous	0.00971	0.623
35	p5_2	Durante la última semana, ¿estuvo cerca de aparatos electrónicos prendidos (celular, Tablet, televisor, videoj	Near switched-on electronic devices while eating, past week (frequency)	1 = Sí, siempre; 2 = Sí, habitualmente; 3 = No, nunca ...	0.00963	0.618

36	p8_11_9	(Seguridad del barrio) ¿Qué problemas relacionados de contaminación o deterioro del entorno identifica usted e	Neighbourhood safety identified as a problem	1 = Sí; 2 = No; 8 = No sabe (NO LEER) ...	0.00946	0.607
37	GRUPO_CIIU	Clasificación Industrial Internacional Uniforme de todas las Actividades Económicas: Grandes grupos (1 dígito)	Economic activity of employer (ISIC, one digit)	-1 = No sabe o No responde; 1 = Agricultura, ganadería, caza, silvicultura y pesca; 2 = Explotación de minas y canteras ...	0.00931	0.598
38	p2_13_2_d	(Sentarse a ver televisión abierta, por cable o satelital) Fin de semana	Weekend hours watching television	continuous	0.00912	0.585
39	p2_13_2_a	(Sentarse a ver series o películas (Ej. Netflix, YouTube, otras plataformas)) Fin de semana	Weekend hours watching series or films on streaming platforms	continuous	0.00910	0.584
40	p8_12_8	(Termitas) En su casa o barrio, ¿ha notado la presencia de…?	Termites noticed in home or neighbourhood	1 = Sí; 2 = No; 8 = No sabe (NO LEER) ...	0.00888	0.570
41	p2_13_1_a	(Sentarse a ver series o películas (Ej. Netflix, YouTube, otras plataformas)) Semana	Weekday hours watching series or films on streaming platforms	continuous	0.00858	0.551
42	p8_3	Cuando tiene problemas… ¿Tiene Ud. alguna persona en quien confiar, pedir ayuda o consejos?	Has someone to trust or ask for help or advice when in trouble	1 = Sí, siempre; 2 = Sí, casi siempre; 3 = Sí, algunas veces ...	0.00857	0.550
43	p5_17	Durante el último mes, ¿ha fumado usted cigarrillos?	Smoked cigarettes during the past month	1 = Sí; 2 = No, dejó de fumar hace menos de 6 meses; 3 = No, dejó de fumar hace más de 6 meses ...	0.00857	0.550
44	quintiles	Quintiles de ingreso total por hogar	Household income quintile	1 = I; 2 = II; 3 = III ...	0.00840	0.539
45	p2_13_1_b_rec	(Usar redes sociales como Instagram, Facebook, TikTok o Twitter) Semana	Weekday hours using social media (categorised)	1 = Menos de 3 horas; 2 = Más de 3 horas y menos de 6; 3 = Más de 6 horas y menos de 9 ...	0.00839	0.539
46	p8_11_1	(Ruidos molestos) ¿Qué problemas relacionados de contaminación o deterioro del entorno identifica usted en su	Annoying noise identified as a neighbourhood problem	1 = Sí; 2 = No; 8 = No sabe (NO LEER) ...	0.00832	0.534
47	p8_9_7	(Si necesito un consejo puedo pedírselo a una persona de mi barrio) ¿Cuán de acuerdo está con las siguientes f	Agreement: if I need advice I can ask someone in my neighbourhood	1 = Muy de acuerdo; 2 = De acuerdo; 3 = Ni de acuerdo ni en desacuerdo ...	0.00829	0.533
48	p_4_11_1	(Cepillo de dientes con pasta dental) Piense en una semana promedio de lunes a domingo. ¿Con qué frecuencia ut	Weekly frequency of tooth brushing with toothpaste	1 = Nunca; 2 = A veces; 3 = Casi siempre ...	0.00804	0.516
49	p6_2_7_a	(Automóvil (asiento trasero)) En los últimos 30 días, ¿ha sido pasajero en los siguientes vehículos: automóvil	Passenger in a car (rear seat) in the past 30 days	1 = Sí; 2 = No	0.00787	0.506
50	p6_4_7_a	(Buses interprovinciales) En los últimos 30 días, ¿ha sido pasajero en los siguientes vehículos: automóvil, mo	Passenger on an inter-provincial bus in the past 30 days	1 = Sí; 2 = No	0.00781	0.501
51	p8_8	En términos generales, ¿cómo evaluaría su barrio o vecindario como un lugar para vivir?	Overall rating of the neighbourhood as a place to live	1 = Muy Malo; 2 = Malo; 3 = Ni bueno, ni malo ...	0.00754	0.484
52	p8_11_2	(Contaminación del aire) ¿Qué problemas relacionados de contaminación o deterioro del entorno identifica usted	Air pollution identified as a neighbourhood problem	1 = Sí; 2 = No; 8 = No sabe (NO LEER) ...	0.00745	0.478
53	p1_9	¿Cuál es su estado conyugal o civil actual?	Current marital or civil status	1 = Casado(a); 2 = Conviviente o pareja sin acuerdo de unión civil; 3 = Conviviente civil (con acuerdo de unión civil) ...	0.00725	0.466
54	p8_9_9	(Creo que mis vecinos podrían ayudarme en caso de una emergencia) ¿Cuán de acuerdo está con las siguientes fra	Agreement: my neighbours could help me in an emergency	1 = Muy de acuerdo; 2 = De acuerdo; 3 = Ni de acuerdo ni en desacuerdo ...	0.00720	0.462
55	p_4_11_2	(Hilo o seda dental) Piense en una semana promedio de lunes a domingo. ¿Con qué frecuencia utilizó los siguien	Weekly frequency of dental-floss use	1 = Nunca; 2 = A veces; 3 = Casi siempre ...	0.00708	0.455
56	p8_9_8	(Creo que estoy de acuerdo con la mayoría de las personas de mi barrio acerca de lo que es importante en la vi	Agreement: I agree with most people in my neighbourhood about what is important in life	1 = Muy de acuerdo; 2 = De acuerdo; 3 = Ni de acuerdo ni en desacuerdo ...	0.00708	0.454
57	p2_15	En general, ¿cuántas horas al día diría que usa habitualmente el celular?	Usual daily hours of mobile-phone use	continuous	0.00666	0.428
58	p8_9_5	(Si tuviera la oportunidad, me gustaría irme de este barrio) ¿Cuán de acuerdo está con las siguientes frases?	Agreement: if I had the chance I would leave this neighbourhood	1 = Muy de acuerdo; 2 = De acuerdo; 3 = Ni de acuerdo ni en desacuerdo ...	0.00649	0.417

59	p_4_11_3	(Enjuague bucal) Piense en una semana promedio de lunes a domingo. ¿Con qué frecuencia utilizó los siguientes	Weekly frequency of mouthwash use	1 = Nunca; 2 = A veces; 3 = Casi siempre ...	0.00647	0.416
60	p11_17_1	(¿Los servicios básicos como luz, agua y gas?) Y, diría que el ingreso mensual del hogar les permite pagar sin	Household income covers basic services (electricity, water, gas)	1 = Sí; 2 = No; 8 = No sabe (NO LEER) ...	0.00642	0.412
61	p8_9_6	(Si la gente de mi barrio planifica algo, yo pensaría que es algo que estamos haciendo juntos y no algo que ot	Agreement: if people in my neighbourhood plan something, I think of it as something we do together	1 = Muy de acuerdo; 2 = De acuerdo; 3 = Ni de acuerdo ni en desacuerdo ...	0.00625	0.401
62	elegibles	Elegibles en hogar	Number of eligible persons in the household	continuous	0.00623	0.400
63	p2_14	Imagínese que en un día cualquiera olvida su celular en casa, ¿el resto del día usted estaría intranquilo, ind	Feeling if the mobile phone were forgotten at home for a day	1 = Intranquilo; 2 = Indiferente; 3 = Aliviado ...	0.00623	0.400
64	p8_9_10	(Yo siento lealtad hacia la gente de mi barrio) ¿Cuán de acuerdo está con las siguientes frases?	Agreement: I feel loyalty towards the people of my neighbourhood	1 = Muy de acuerdo; 2 = De acuerdo; 3 = Ni de acuerdo ni en desacuerdo ...	0.00617	0.396
65	p8_9_13	(Planeo seguir viviendo en este barrio por varios años) ¿Cuán de acuerdo está con las siguientes frases?	Agreement: I plan to keep living in this neighbourhood for several years	1 = Muy de acuerdo; 2 = De acuerdo; 3 = Ni de acuerdo ni en desacuerdo ...	0.00613	0.394
66	p9_1_3	(Le ha amenazado) Durante los últimos 12 meses, le ha pasado que una persona conocida o desconocida…	Threatened by someone, past 12 months	1 = Sí; 2 = No; 88 = No sabe (NO LEER) ...	0.00594	0.382
67	p8_1_1	(Juntas de vecinos u otra organización territorial (comité de aguas, comité de allegados, otros).) Participa U	Participates in a neighbourhood council or other territorial organisation	1 = Sí; 2 = No; 8 = No sabe (NO LEER) ...	0.00591	0.379
68	p8_13	¿Qué tan seguro/a se siente caminando solo/a en su barrio cuando ya está oscuro?	How safe respondent feels walking alone in the neighbourhood after dark	1 = Muy inseguro; 2 = Un poco inseguro; 3 = Medianamente seguro ...	0.00589	0.378
69	p2_13_1_d	(Sentarse a ver televisión abierta, por cable o satelital) Semana	Weekday hours watching broadcast, cable or satellite television	continuous	0.00581	0.373
70	p2_13_2_b	(Usar redes sociales como Instagram, Facebook, TikTok o Twitter) Fin de semana	Weekend hours using social media	continuous	0.00516	0.331
71	p10_6	El material predominante en las paredes exteriores de la vivienda es:	Predominant material of the dwelling's exterior walls	1 = Hormigón armado, piedra; 2 = Ladrillo; 3 = Paneles estructurales, bloques (Prefabricado) ...	0.00515	0.330
72	p10_10	¿Cuántos dormitorios (uso exclusivo para dormir) tiene la vivienda?	Number of bedrooms in the dwelling	8888 = No sabe; 9999 = No responde	0.00510	0.327
73	p8_9_4	(Las amistades y relaciones que tengo con otras personas de mi barrio significan mucho para mí) ¿Cuán de acuer	Agreement: friendships with people in my neighbourhood mean a lot to me	1 = Muy de acuerdo; 2 = De acuerdo; 3 = Ni de acuerdo ni en desacuerdo ...	0.00485	0.311
74	p8_11_4	(Contaminación del suelo) ¿Qué problemas relacionados de contaminación o deterioro del entorno identifica uste	Soil pollution identified as a neighbourhood problem	1 = Sí; 2 = No; 8 = No sabe (NO LEER) ...	0.00476	0.306
75	p10_9	¿Cuá es el principal combustible usado para calefaccionar este hogar?	Main heating fuel in the household	1 = Gas natural o por cañería; 2 = Gas licuado; 3 = Parafina ...	0.00475	0.305
76	p8_9_15	(Rara vez mis vecinos me visitan en mi casa) ¿Cuán de acuerdo está con las siguientes frases?	Agreement: my neighbours rarely visit me at home	1 = Muy de acuerdo; 2 = De acuerdo; 3 = Ni de acuerdo ni en desacuerdo ...	0.00471	0.303
77	p5_1_2_b	(Almuerzo) ¿Y con quién comparte esta comida la mayoría de las veces?	With whom lunch is usually shared	1 = Solo; 2 = Con familiares; 3 = Con amigos ...	0.00467	0.300
78	p8_1_7	(¿Alguna otra organización?) Participa Ud. activa o frecuentemente en las siguientes organizaciones	Participates in any other organisation	1 = Sí; 2 = No; 8 = No sabe (NO LEER) ...	0.00457	0.294
79	p8_12_5	(Cucarachas) En su casa o barrio, ¿ha notado la presencia de…?	Cockroaches noticed in home or neighbourhood	1 = Sí; 2 = No; 8 = No sabe (NO LEER) ...	0.00454	0.292
80	p8_9_12	(Estoy dispuesto a colaborar con otras personas para mejorar mi barrio) ¿Cuán de acuerdo está con las siguient	Agreement: I am willing to work with others to improve my neighbourhood	1 = Muy de acuerdo; 2 = De acuerdo; 3 = Ni de acuerdo ni en desacuerdo ...	0.00454	0.291
81	p8_10_14	(Falta de acceso a Internet) ¿Qué problemas de infraestructura y equipamiento identifica usted en su barrio o	Lack of internet access identified as a neighbourhood problem	1 = Sí; 2 = No; 8 = No sabe (NO LEER) ...	0.00451	0.289
82	p8_9_17	(Regularmente me paro a conversar con las personas en mi barrio) ¿Cuán de acuerdo está con las siguientes fras	Agreement: I regularly stop to talk with people in my neighbourhood	1 = Muy de acuerdo; 2 = De acuerdo; 3 = Ni de acuerdo ni en desacuerdo ...	0.00437	0.281
83	p10_7	El material predominante en el piso de la vivienda es:	Predominant flooring material of the dwelling	1 = Parquet, madera, piso flotante o similar; 2 =	0.00407	0.261

				Cerámica, flexit o similar; 3 = Alfombra o cubrepiso ...		
84	p5_3	¿Dónde compra preferentemente los alimentos frescos, como frutas y verduras, pescados, huevos, etc.?	Where fresh food is usually bought	1 = Ferias y mercados; 2 = Supermercados; 3 = Verdulerías y fruterías cercanos a su hogar ...	0.00403	0.259
85	p1_11	¿Tiene hijos y/o hijas?	Has children	1 = Sí; 2 = No; 8 = No sabe (NO LEER) ...	0.00398	0.255
86	p8_6	¿Tiene mascotas?	Has pets	1 = Sí; 2 = No; 8 = No sabe (NO LEER) ...	0.00398	0.255
87	p2_13_1_d_rec	(Sentarse a ver televisión abierta, por cable o satelital) Semana	Weekday hours watching television (categorised)	1 = Menos de 3 horas; 2 = Más de 3 horas y menos de 6; 3 = Más de 6 horas y menos de 9 ...	0.00382	0.245
88	p6_1_7_a	(Automóvil (asiento delantero)) En los últimos 30 días, ¿ha sido pasajero en los siguientes vehículos: automóv	Passenger in a car (front seat) in the past 30 days	1 = Sí; 2 = No	0.00372	0.239
89	k01	Personas en hogar	Number of persons in the household	continuous	0.00369	0.237
90	p1_8	En Chile, la ley reconoce diez pueblos indígenas, ¿pertenece usted o es descendiente de alguno de ellos?	Belongs to or descends from an indigenous people	1 = Aimara; 2 = Rapa-Nui o Pascuenses; 3 = Quechua ...	0.00366	0.235
91	p8_1_3	(Organización religiosa o de iglesia.) Participa Ud. activa o frecuentemente en las siguientes organizaciones	Participates in a religious or church organisation	1 = Sí; 2 = No; 8 = No sabe (NO LEER) ...	0.00359	0.231
92	p5_16_1	(…En su hogar) ¿Está Ud. expuesto al humo de tabaco?	Exposed to tobacco smoke at home	1 = Sí; 2 = No; 8 = No sabe (NO LEER) ...	0.00339	0.218
93	p8_9_2	(Siento que pertenezco a este barrio) ¿Cuán de acuerdo está con las siguientes frases?	Agreement: I feel I belong to this neighbourhood	1 = Muy de acuerdo; 2 = De acuerdo; 3 = Ni de acuerdo ni en desacuerdo ...	0.00337	0.216
94	p5_6	Durante los últimos 7 días, ¿en cuántos días hizo actividades físicas moderadas tales como transportar pesos l	Days of moderate physical activity in the past 7 days	continuous	0.00330	0.212
95	p8_9_11	(Con mis vecinos nos prestamos cosas y nos hacemos favores) ¿Cuán de acuerdo está con las siguientes frases?	Agreement: my neighbours and I lend each other things and do favours	1 = Muy de acuerdo; 2 = De acuerdo; 3 = Ni de acuerdo ni en desacuerdo ...	0.00317	0.204
96	p10_4_o1	(Falta o poca cantidad de agua potable) ¿Qué problemas de infraestructura básica identifica usted con su vivie	Lack or shortage of drinking water in the dwelling	1 = Sí; 2 = No	0.00313	0.201
97	p8_9_18	(Vivir en este barrio me da una sensación de comunidad) ¿Cuán de acuerdo está con las siguientes frases?	Agreement: living in this neighbourhood gives me a sense of community	1 = Muy de acuerdo; 2 = De acuerdo; 3 = Ni de acuerdo ni en desacuerdo ...	0.00313	0.201
98	p8_10_9	(Iluminación deficiente) ¿Qué problemas de infraestructura y equipamiento identifica usted en su barrio o loca	Poor street lighting identified as a neighbourhood problem	1 = Sí; 2 = No; 8 = No sabe (NO LEER) ...	0.00308	0.198
99	valor_ipaq	Cuestionario internacional de actividad física (IPAQ)	Physical-activity category (IPAQ)	1 = Inactivo; 2 = Minimamente activo; 3 = Activo HEPA	0.00300	0.192
100	p8_10_4	(Falta de centros comunitarios, lugares de reunión social o recreación) ¿Qué problemas de infraestructura y eq	Lack of community centres or meeting places identified as a neighbourhood problem	1 = Sí; 2 = No; 8 = No sabe (NO LEER) ...	0.00296	0.190
101	p9_1_2	(Le ha controlado o celado) Durante los últimos 12 meses, le ha pasado que una persona conocida o desconocida…	Controlled or subjected to jealousy by someone, past 12 months	1 = Sí; 2 = No; 88 = No sabe (NO LEER) ...	0.00278	0.178
102	p5_20	¿Ha tomado Ud. alcohol alguna vez en su vida?	Has ever drunk alcohol	1 = Sí; 2 = No; 8 = No sabe ...	0.00264	0.169
103	macrozona	Macrozona	Macro-zone of residence	1 = Norte; 2 = Centro; 3 = Sur ...	0.00254	0.163
104	p8_12_4	(Pulgas) En su casa o barrio, ¿ha notado la presencia de…?	Fleas noticed in home or neighbourhood	1 = Sí; 2 = No; 8 = No sabe (NO LEER) ...	0.00254	0.163
105	p8_9_1	(En general me siento muy cómodo de vivir en este barrio) ¿Cuán de acuerdo está con las siguientes frases?	Agreement: in general I feel very comfortable living in this neighbourhood	1 = Muy de acuerdo; 2 = De acuerdo; 3 = Ni de acuerdo ni en desacuerdo ...	0.00248	0.159
106	p10_8	¿Cuál es el principal combustible usado para cocinar en este hogar?	Main cooking fuel in the household	1 = Gas natural o por cañería; 2 = Gas licuado; 3 = Parafina ...	0.00241	0.155
107	p2_13_2_c	(Sentarse a jugar videojuegos) Fin de semana	Weekend hours playing video games	continuous	0.00232	0.149
108	p8_10_11	(Falta o deficiencia del transporte público) ¿Qué problemas de infraestructura y equipamiento identifica usted	Lack or deficiency of public transport identified as a neighbourhood problem	1 = Sí; 2 = No; 8 = No sabe (NO LEER) ...	0.00231	0.148
109	sum_comidas	Suma de comidas al dia en una semana promedio	Number of meals per day in an average week	continuous	0.00202	0.129

110	p8_10_1	(Falta de plazas, áreas verdes, otras) ¿Qué problemas de infraestructura y equipamiento identifica usted en su	Lack of squares or green areas identified as a neighbourhood problem	1 = Sí; 2 = No; 8 = No sabe (NO LEER) ...	0.00200	0.128
111	p8_10_10	(Pavimentación insuficiente o en mal estado.) ¿Qué problemas de infraestructura y equipamiento identifica uste	Insufficient or poor paving identified as a neighbourhood problem	1 = Sí; 2 = No; 8 = No sabe (NO LEER) ...	0.00197	0.127
112	p5_1_1_a	(Desayuno) Respecto a sus tiempos de comida, y considerando una semana promedio de lunes a viernes, ¿la mayor	Where breakfast is usually eaten on weekdays	1 = Casa; 2 = Trabajo; 3 = Otro lugar ...	0.00195	0.125
113	p8_10_7	(Falta de sombreadoras o toldos en plazas) ¿Qué problemas de infraestructura y equipamiento identifica usted e	Lack of shade structures in squares identified as a neighbourhood problem	1 = Sí; 2 = No; 8 = No sabe (NO LEER) ...	0.00195	0.125
114	p10_1	¿Cómo obtiene el agua potable?	Source of drinking water	1 = Sistema de agua potables rural (APR); 2 = Sistema de distribución de agua potable (con tratamiento); 3 = Directamente desde la fuente natural (sin tratamiento)	0.00194	0.125
115	p5_1_4_a	(Cena) Respecto a sus tiempos de comida, y considerando una semana promedio de lunes a viernes, ¿la mayor part	Where dinner is usually eaten on weekdays	1 = Casa; 2 = Trabajo; 3 = Otro lugar ...	0.00190	0.122
116	p8_10_5	(Falta de locales comerciales) ¿Qué problemas de infraestructura y equipamiento identifica usted en su barrio	Lack of shops identified as a neighbourhood problem	1 = Sí; 2 = No; 8 = No sabe (NO LEER) ...	0.00189	0.121
117	p10_5	Su casa, ¿qué tipo de vivienda es?	Type of dwelling	1 = Casa; 2 = Departamento en edificio; 3 = Piezas o vivienda conventillo ...	0.00188	0.121
118	p8_10_2	(Falta de infraestructura deportiva (canchas, gimnasios, pistas, circuitos, máquinas para hacer deporte en las	Lack of sports facilities identified as a neighbourhood problem	1 = Sí; 2 = No; 8 = No sabe (NO LEER) ...	0.00177	0.114
119	p8_10_12	(Falta o deficiencia de limpieza de calles o veredas) ¿Qué problemas de infraestructura y equipamiento identif	Lack of street or pavement cleaning identified as a neighbourhood problem	1 = Sí; 2 = No; 8 = No sabe (NO LEER) ...	0.00164	0.105
120	p8_1_6	(Centros de padres y apoderados.) Participa Ud. activa o frecuentemente en las siguientes organizaciones	Participates in a parents' association	1 = Sí; 2 = No; 8 = No sabe (NO LEER) ...	0.00159	0.102
121	p8_10_6	(Falta de juegos infantiles) ¿Qué problemas de infraestructura y equipamiento identifica usted en su barrio o	Lack of children's playgrounds identified as a neighbourhood problem	1 = Sí; 2 = No; 8 = No sabe (NO LEER) ...	0.00149	0.096
122	p8_12_10	(Murciélagos) En su casa o barrio, ¿ha notado la presencia de…?	Bats noticed in home or neighbourhood	1 = Sí; 2 = No; 8 = No sabe (NO LEER) ...	0.00148	0.095
123	p5_1_3_b	(Once) ¿Y con quién comparte esta comida la mayoría de las veces?	With whom the afternoon meal (once) is usually shared	1 = Solo; 2 = Con familiares; 3 = Con amigos ...	0.00147	0.094
124	p5_4	Durante los últimos 7 días, ¿en cuantos realizó actividades físicas intensas tales como levantar cosas pesadas	Days of vigorous physical activity in the past 7 days	continuous	0.00140	0.090
125	p6_3_4_a	(Bicicleta) En los últimos 30 días, ¿ha conducido uno de los siguientes vehículos: automóvil, motocicleta y bi	Rode a bicycle in the past 30 days	1 = Sí; 2 = No	0.00127	0.081
126	p8_10_8	(Falta de puntos de reciclaje o servicios de reciclaje) ¿Qué problemas de infraestructura y equipamiento ident	Lack of recycling points identified as a neighbourhood problem	1 = Sí; 2 = No; 8 = No sabe (NO LEER) ...	0.00118	0.076
127	p2_13_1_c	(Sentarse a jugar videojuegos) Semana	Weekday hours playing video games	continuous	0.00111	0.071
128	p8_11_3	(Contaminación del agua) ¿Qué problemas relacionados de contaminación o deterioro del entorno identifica usted	Water pollution identified as a neighbourhood problem	1 = Sí; 2 = No; 8 = No sabe (NO LEER) ...	0.00104	0.067
129	p8_10_3	(Falta de ciclovías) ¿Qué problemas de infraestructura y equipamiento identifica usted en su barrio o localida	Lack of cycle lanes identified as a neighbourhood problem	1 = Sí; 2 = No; 8 = No sabe (NO LEER) ...	0.00098	0.063
130	p5_16_2	(…En su lugar de trabajo o estudio) ¿Está Ud. expuesto al humo de tabaco?	Exposed to tobacco smoke at work or place of study	1 = Sí; 2 = No; 8 = No sabe (NO LEER) ...	0.00093	0.060
131	p5_1_2_a	(Almuerzo) Respecto a sus tiempos de comida, y considerando una semana promedio de lunes a viernes, ¿la mayor	Where lunch is usually eaten on weekdays	1 = Casa; 2 = Trabajo; 3 = Otro lugar ...	0.00084	0.054
132	p8_1_2	(Club deportivo o recreativo (de fútbol, de pesca, de juegos de mesa, otros).) Participa Ud. activa o frecuent	Participates in a sports or recreational club	1 = Sí; 2 = No; 8 = No sabe (NO LEER) ...	0.00077	0.049

133	p3_1	Usted actualmente se identifica como:	Self-identified sexual orientation	1 = Gay (atracción de un hombre hacia otro hombre); 2 = Lesbiana (atracción de una mujer hacia otra mujer); 3 = Bisexual (atracción hacia hombres y mujeres) ...	0.00076	0.049
134	p8_12_7	(Chinches) En su casa o barrio, ¿ha notado la presencia de…?	Bedbugs noticed in home or neighbourhood	1 = Sí; 2 = No; 8 = No sabe (NO LEER) ...	0.00071	0.046
135	p5_1_3_a	(Once) Respecto a sus tiempos de comida, y considerando una semana promedio de lunes a viernes, ¿la mayor part	Where the afternoon meal (once) is usually eaten on weekdays	1 = Casa; 2 = Trabajo; 3 = Otro lugar ...	0.00070	0.045
136	p6_3	En los últimos 6 meses, ¿ha tenido Ud. algún accidente o siniestro de tránsito?	Road-traffic accident in the past 6 months	1 = Sí, pero no hizo falta atención médica; 2 = Sí y requirió atención médica pero no hospitalización; 3 = Sí y requirió atención médica y hospitalización ...	0.00050	0.032
137	p6_2_4_a	(Motocicleta) En los últimos 30 días, ¿ha conducido uno de los siguientes vehículos: automóvil, motocicleta y	Rode a motorcycle as driver in the past 30 days	1 = Sí; 2 = No	0.00046	0.029
138	p6_6	Durante los últimos 30 días, ¿cuántas veces anduvo en un vehículo motorizado cuyo conductor haya bebido 1 o má	Times as passenger of a driver who had been drinking, past 30 days	continuous	0.00044	0.028
139	once	Toma once en una semana promedio	Has the afternoon meal (once) in an average week	1 = Toma once; 2 = No toma once; 3 = No sabe/no responde	0.00039	0.025
140	area	Zona	Urban or rural residence	1 = Urbano; 2 = Rural	0.00036	0.023
141	desayuno	Desayuna en una semana promedio	Has breakfast in an average week	1 = Desayuna; 2 = No desayuna; 3 = No sabe o No responde	0.00033	0.021
142	p2_13_1_c_rec	(Sentarse a jugar videojuegos) Semana	Weekday hours playing video games (categorised)	1 = Menos de 3 horas; 2 = Más de 3 horas y menos de 6; 3 = Más de 6 horas y menos de 9 ...	0.00024	0.015
143	p1_5	¿Cuál es su nacionalidad?	Nationality	1 = Chile; 2 = Otro; 9 = No responde (NO LEER)	0.00011	0.007
144	p10_3	¿Su vivienda se provee de agua potable a través de camiones aljibes?	Drinking water supplied by tanker truck	1 = Sí; 2 = No	0.00011	0.007
145	p6_3_7_a	(Motocicleta) En los últimos 30 días, ¿ha sido pasajero en los siguientes vehículos: automóvil, motocicleta, b	Passenger on a motorcycle in the past 30 days	1 = Sí; 2 = No	0.00005	0.003
146	p5_19_1	(…En su hogar) ¿Está Ud. expuesto al humo del cigarrillo electrónico como vaporizadores u otros similares?	Exposed to e-cigarette vapour at home	1 = Sí; 2 = No; 8 = No sabe (NO LEER) ...	0.00004	0.003
147	p10_2	¿Cuál es el sistema de distribución del agua en la vivienda?	Water distribution system in the dwelling	1 = Con llave dentro de la vivienda.; 2 = Con llave dentro del sitio, pero fuera de la vivienda.; 3 = No tiene sistema, la acarrea.	0.00003	0.002
148	p5_19_2	(…En su lugar de trabajo o estudio) ¿Está Ud. expuesto al humo del cigarrillo electrónico como vaporizadores u	Exposed to e-cigarette vapour at work or place of study	1 = Sí; 2 = No; 8 = No sabe (NO LEER) ...	0.00000	0.000
149	p8_12_9	(Vinchucas) En su casa o barrio, ¿ha notado la presencia de…?	Kissing bugs (vinchucas) noticed in home or neighbourhood	1 = Sí; 2 = No; 8 = No sabe (NO LEER) ...	0.00000	0.000
150	p11_1	¿La persona que responderá este módulo corresponde con la persona que respondió el resto de la encuesta?	Household-income module answered by the same respondent	1 = Sí; 2 = No; 8 = No sabe (NO LEER) ...	0.00000	0.000
151	almuerzo	Almuerzo en una semana promedio	Has lunch in an average week	1 = Almuerza; 2 = No almuerza; 3 = No sabe o No responde	0.00000	0.000

Table S3. Per-split robustness

The entire pipeline (age-spline residualisation, model fitting and evaluation) was repeated on five independent 70/30 splits grouped by primary sampling unit, each with a different random seed.

Split	R^2 age stage	R^2 residual	MAE	RMSE
seed 42	0.232	+0.1166	1.1736	1.5928
seed 101	0.218	+0.1231	1.2243	1.7052
seed 202	0.244	+0.1102	1.1909	1.6313
seed 303	0.245	+0.1440	1.1366	1.5343

seed 404	0.261	+0.1323	1.1501	1.5514
Mean ± SD	—	0.1252 ± 0.0133	—	—

Coefficient of variation of test R^2 across splits: 10.6% of the mean. Mean pairwise Spearman correlation of the importance ranking: 0.915. Six of the ten leading determinants appeared in the top ten of all five splits. Internal metric coherence was verified: $R^2 = 1 - RMSE^2/variance$ held to within 0.002 for all 17 algorithms, RMSE exceeded MAE in every case, and the ordering by R^2 was identical to the ordering by RMSE.

Table S4. Principal-component sensitivity analysis

Dimensionality reduction was tested as an alternative to modelling the original variables. Both rows are cross-validated values for the random forest on the same folds, and are therefore comparable with each other but not with the random-forest test R^2 reported in the main text.

Representation	Features	Algorithm	R^2 (cross-validated)	SHAP interpretability
Original variables	151	Random forest	0.1206	Direct
Principal components	45	Random forest	0.0961	Indirect

Weighted PCA on the standardised determinants showed no low-dimensional structure: the first component explained 5.6% of variance, the first ten 26.4%, and 86 components were required to reach 80%. SHAP applied to components identifies which component matters but not which variable, because each component mixes dozens of items.

Table S5. Domain aggregation

Domains were assigned by variable prefix before attribution was computed. Domain attribution is the sum of member attributions. All eight domains are shown so that attribution sums to the whole model rather than to a selected subset.

Domain	Variables, n	Summed mean |SHAP|	% of total attribution
Social isolation and support	30	0.360	23.1
Neighbourhood environment	45	0.326	20.9
Socio-economic position	10	0.316	20.3
Demographics	10	0.223	14.3
Health behaviours	36	0.186	12.0
Violence and discrimination	5	0.073	4.7
Other	6	0.038	2.5
Mobility and transport	9	0.034	2.2

"Other" comprises household-composition and area variables not assignable to a substantive domain. Percentages sum to 100 within rounding. Socio-economic position achieved the highest attribution per variable, followed by demographics and violence and discrimination; the neighbourhood domain distributed its contribution across many.

Table S6. Stability of domain attribution across splits

The full pipeline (age-spline residualisation, random forest fitting and SHAP attribution) was repeated on each of the five independent PSU-grouped splits, and domain shares recomputed. This tests whether the headline ordering depends on a single partition.

Domain	Split 1	Split 2	Split 3	Split 4	Split 5	Mean ± SD
Social isolation and support	23.1	22.7	24.8	22.6	21.8	23.0 ± 1.1
Neighbourhood environment	20.9	20.8	21.0	22.3	22.4	21.5 ± 0.8
Socio-economic position	20.3	16.9	21.2	15.3	19.4	18.6 ± 2.5
Demographics	14.3	16.9	10.5	15.4	11.5	13.7 ± 2.7
Health behaviours	12.0	11.6	12.6	13.3	12.8	12.5 ± 0.7
Violence and discrimination	4.7	5.6	4.6	5.1	6.8	5.4 ± 0.9
Other	2.5	2.4	2.5	2.5	2.1	2.4 ± 0.2
Mobility and transport	2.2	3.1	2.8	3.6	3.2	3.0 ± 0.5

Values are the percentage of total SHAP attribution accounted for by each domain. Each of the three social and environmental domains exceeded health behaviours in all five splits, and the ordering of the top two domains was preserved throughout. As a further check, domain shares were also computed from impurity-based random-forest importances; these placed health behaviours substantially higher than SHAP did, a discrepancy consistent with the known bias of impurity importance towards continuous, high-cardinality predictors, of which the behavioural block contains several. SHAP is reported as the primary measure for this reason.

Table S7. Characteristics of respondents included in and excluded from the analytic sample

Design-weighted values for the 12,070 respondents with complete data on the outcome and the core demographic and behavioural indicators (Section 2.3 of the main text) and for the 4,520 respondents excluded by the complete-case rule. Values are weighted percentages unless the row label indicates a mean. Outcome rows use the 4,422 excluded respondents for whom the condition count was observed. Exclusions were due mainly to a missing weekday sleep-duration item (66% of excluded respondents) and to missing weight or height (40%); the two overlap. Only 2% of exclusions were due to a missing outcome.

Characteristic	Included (n = 12,070)	Excluded (n = 4,520)
Age, mean (years)	44.2	45.0
Aged 65 years or older	15.7	19.1
Women	50.0	54.4
Tertiary education	39.9	32.4
Rural residence	10.9	13.2
Self-rated economic situation poor	8.6	10.1
Income does not cover food all month	13.2	17.7
Often lacks companionship	11.0	13.1
Bad odours in neighbourhood	30.5	31.6
Made to fear assault or abuse	10.0	10.7
Experienced discrimination	7.0	8.2
Chronic conditions, mean (n), where observed	1.66	1.74
Multimorbidity, where observed	40.1	40.3

Table S8. Domain attribution in the sensitivity analyses

Percentage of total mean absolute SHAP attribution received by each domain on the held-out test set of the seed-42 split, under the main specification and four alternatives (Section 2.7 of the main text). All columns were produced with the same random-forest configuration (300 trees, minimum leaf size 20), chosen so that repeated SHAP computation was tractable; the first column is therefore identical to Table S5 and to Table 4 of the main text, and provides the like-with-like reference for the others. a Includes continuous age (24.0%) and age band (12.6%). b R^2 on the raw condition count, comparable with the chained two-stage figure of 32.2% rather than with the other columns.

Domain	Age-residualised, age band retained	No residualisation: all variables	No residualisation: age variables excluded, renormalised	Age-residualised, age band removed	Age-residualised, inclusion-probability weighted
Social isolation and support	23.1	12.8	20.1	23.8	23.3
Neighbourhood environment	20.9	11.8	18.7	20.9	20.9
Socio-economic position	20.3	17.6	27.8	20.0	19.7
Demographics	14.3	43.7 a	11.2	12.5	14.6
Health behaviours	12.0	8.0	12.6	12.6	12.1
Violence and discrimination	4.7	2.5	3.9	5.2	5.0
Mobility and transport	2.2	2.1	3.3	2.6	2.2
Other	2.5	1.5	2.4	2.5	2.2
Test R^2	0.117	0.320 b	0.320 b	0.114	0.118

Table S9. Design-weighted logistic model of inclusion in the analytic sample

Standardised coefficients (log-odds per standard deviation) of the model used to derive inclusion-probability weights. Fitted inclusion probabilities among included respondents ranged from 0.46 to 0.87 (median 0.76). Inclusion rises with age up to middle age and falls thereafter, and rises with education; sex, income quintile and rural residence contribute little.

Variable	Standardised coefficient
Age (years)	0.79
Age squared	−0.80
Female sex	−0.08
Educational level	0.22
Household income quintile	0.02
Rural residence	−0.04
Region	0.10

Table S10. Domain attribution under four normalisations

The domains contain between 5 and 45 variables, so a domain with more members has more opportunity to accumulate attribution. The table reports the summed share used in the main text alongside three size-

independent alternatives: the mean attribution of a domain's members, their median attribution, and the sum restricted to the five leading members of each domain, five being the size of the smallest domain, renormalised to 100%. The rank of each domain under each normalisation is given in parentheses; 6= denotes a tie. Health behaviours ranks fifth of eight on summed attribution and on the top-five restriction and seventh of eight per variable, below every other substantive domain; the domains that lead do so with few strong members rather than with many weak ones. Under the median it is indistinguishable from the neighbourhood environment, the only domain it reaches.

Domain	Variables, n	Summed, % of total	Mean per variable (rank)	Median per variable (rank)	Top five members, % (rank)
Social isolation and support	30	23.1	0.012 (4)	0.006 (4)	20.2 (2)
Neighbourhood environment	45	20.9	0.007 (5)	0.003 (6=)	15.0 (4)
Socio-economic position	10	20.3	0.032 (1)	0.013 (1)	24.6 (1)
Demographics	10	14.3	0.022 (2)	0.004 (5)	19.4 (3)
Health behaviours	36	12.0	0.005 (7)	0.003 (6=)	7.7 (5)
Violence and discrimination	5	4.7	0.015 (3)	0.012 (2)	6.7 (6)
Other	6	2.5	0.006 (6)	0.007 (3)	3.4 (7)
Mobility and transport	9	2.2	0.004 (8)	0.001 (8)	3.0 (8)

Table S11. Missing-data profile and sensitivity

Missingness of the 151 determinants in the analytic sample, measured after recoding non-response sentinels and before median imputation.

Quantity	Value
Cells imputed, all determinants	1.6% of the analytic matrix
Determinants with no missing value	16 of 151
Median missingness per determinant	0.6%
Determinants above 5% missing	13
Highest missingness	13.9% (main heating fuel of the household)
Mean missingness, socio-economic block	3.2%
Mean missingness, health-behaviour block	3.1%
Mean missingness, violence and discrimination block	0.3%

Domain attribution when the influence of imputation is tested directly. Adding a binary indicator for each of the 13 determinants above 5% missingness lets the model use the fact that a value was imputed; dropping those determinants removes the imputed values altogether, at the cost of removing the two leading members of the behavioural block (daily sitting hours and minutes), so the 7.6% in that row is a lower bound for health behaviours rather than an unbiased estimate. Values are percentages of total attribution on the held-out test set of the seed-42 split, under the configuration described in Table S8.

Specification	Test R^2	Social isolation	Neighbourhood	Socio-economic	Demographics	Health behaviours	Violence
Main analysis (median imputation)	0.117	23.1	20.9	20.3	14.3	12.0	4.7
Missingness indicators added	0.115	23.3	20.7	19.9	14.1	12.2	5.1
13 determinants dropped	0.111	25.5	21.4	19.1	15.3	7.6	5.6

Table S12. Leading neighbourhood problems by household income quintile

Weighted percentage of respondents reporting each problem, by quintile of total household income (I lowest to V highest), with the ratio between the lowest and highest quintile and the largest absolute Spearman correlation between the item and any variable in the socio-economic block. The items are patterned by income but share less than 1% of their variance with any socio-economic measure available in the survey, so they are not interchangeable with it.

Neighbourhood problem	I	II	III	IV	V	I/V ratio	Max |rho| with socio-economic block
Bad odours	33.7	28.3	29.2	19.1	17.3	1.95	0.090
Flies in home or neighbourhood	61.6	56.4	58.1	53.2	49.8	1.24	0.079
Poor drainage, rain and flooding	21.7	19.7	20.8	19.1	16.7	1.30	0.079
Vibrations from traffic or transport	26.9	21.9	24.2	21.9	20.1	1.34	0.052

Table S13. Domain attribution under grouped and survey-weighted measures

Domain attribution of the fixed random forest on the held-out test set of the seed-42 split, computed in a re-run of the main specification for this revision; its summed shares differ from Table 4 of the main text by at most 0.5 percentage points. Grouped SHAP sums the signed SHAP values of a domain's members for each respondent before taking the absolute value and averaging, so that opposing contributions within a domain cancel. Grouped permutation is the mean loss of weighted test R^2 (standard deviation in parentheses) when all members of a domain are permuted jointly, over 20 permutations; the reference test R^2 is 0.117. Health behaviours is sixth of eight under grouped SHAP and fifth under grouped permutation, below each social and environmental domain under every measure.

**Domain**	**Summed mean |SHAP|, %**	**Summed, survey-weighted, %**	**Grouped SHAP, %**	**Grouped SHAP, survey-weighted, %**	**Grouped permutation, loss of test R^2**
Social isolation and support	23.6	22.8	21.1	20.2	0.040 (0.006)
Neighbourhood environment	20.4	20.8	16.7	17.4	0.025 (0.006)
Socio-economic position	19.9	18.8	22.4	20.4	0.042 (0.006)
Demographics	13.9	14.1	19.3	19.3	0.027 (0.005)
Health behaviours	11.9	12.4	7.7	8.5	0.011 (0.003)
Violence and discrimination	5.2	5.9	8.3	9.4	0.010 (0.003)
Mobility and transport	2.6	2.7	2.6	2.8	0.002 (0.001)
Other	2.4	2.5	2.0	2.0	0.001 (0.001)

Table S14. Nested hyperparameter tuning of the penalised linear models and tree ensembles

Nested cross-validation within the training set of the seed-42 split: for each of five PSU-grouped outer folds, a three-fold PSU-grouped inner search selected the settings, and the outer fold scored the selected model. Selected settings are those chosen by the same search on the full training set, refitted and scored on the held-out test set. All fits used survey weights. a Ridge penalties are on the scale of the survey-weighted loss, which is why they are large. b Selected at the edge of the grid; for LightGBM, extending the grid to the values shown raised the nested R^2 from 0.129 to 0.132. c Five-fold cross-validation without nesting, as in Table 3. Algorithms not listed kept the fixed settings of Table 3 and were not used for attribution.

**Algorithm**	**Search space**	**Selected settings**	**Nested CV R^2**	**Test R^2**
Ridge	alpha from 10^0 to 10^8, 17 values on a log scale	alpha = 3.2 × 10^6 a	0.109	0.114
Lasso	alpha ∈ {0.001, 0.003, 0.005, 0.01, 0.015, 0.02, 0.03, 0.05, 0.1}	alpha = 0.02	0.110	0.113
Elastic net	alpha ∈ {0.005, 0.01, 0.02, 0.04, 0.08, 0.15} × l1 ratio ∈ {0.1, 0.5, 0.9}	alpha = 0.04, l1 ratio = 0.5	0.110	0.113
Random forest	300 trees; minimum leaf ∈ {2, 5, 10, 20, 40} × features per split ∈ {0.1, 0.2, 1/3, 0.5}	leaf 5, 0.2	0.125	0.122
Extra Trees	300 trees; minimum leaf ∈ {5, 10, 20, 40} × features per split ∈ {0.2, 1/3, 0.5}	leaf 5, 0.2 b	0.123	0.115
LightGBM	learning rate ∈ {0.005, 0.01, 0.03} × leaves ∈ {7, 15, 31, 63} × trees ∈ {300, 600, 1200}; minimum child samples 40, row subsample	rate 0.005, 63 leaves, 1200 trees b	0.132	0.135

	0.8, feature subsample 0.5			
Histogram gradient boosting	learning rate ∈ {0.02, 0.05} × maximum leaf nodes ∈ {7, 15, 31}; 400 iterations, minimum leaf 40	rate 0.02, 15 leaf nodes	0.116	0.124
Gradient boosting	learning rate ∈ {0.02, 0.05} × depth ∈ {2, 3}; 300 trees, subsample 0.8, feature subsample 0.5	rate 0.05, depth 3	0.110	0.117
Random forest, fixed settings (Table 3)	300 trees, minimum leaf 20, one third of features per split; not searched	—	0.121 c	0.117

Supplementary methods note

Outcome construction. The chronic-condition count was built from 19 individual items (p4_6_1_a to p4_6_1_s) rather than the survey-derived categorisation, which is top-coded at "two or more" and discards the upper tail. Responses of "does not know" or "does not answer" were treated as missing and respondents missing more than two items were excluded. The epilepsy item is administered only to a filtered subgroup (n = 228) and was coded as absent when not applicable; treating it as missing would have excluded 16,362 respondents.

Sentinel codes. Non-response codes vary by item (7/8/9, 77/88/99, 777/888/999, 7777/8888/9999). They were recoded as missing wherever they accounted for less than 35% of an item's responses, including in binary items, where a rule requiring more than three distinct values would miss the pattern {1, 2, 9} and leave the sentinel in the data. Standardised, such cases sit roughly 120 standard deviations from the mean; tree ensembles are robust to this but linear models are not. In the physical-activity items the code 7777 denotes zero days rather than non-response and was recoded as 0; treating it as missing would have removed the 61.5% of respondents reporting no moderate-to-vigorous activity.

Because some items have genuine categories coded 7 to 9, this rule also treated a small number of valid answers as missing, for example widowed marital status, seven days of moderate activity, seven to nine hours of sitting and the "does not heat" category of the heating-fuel item (0.7% of the cells of the analytic matrix, across 26 determinants). The stricter rule used in the sensitivity analysis treats as missing only codes labelled "does not know" or "does not answer" in the questionnaire, together with unlabelled codes such as 88, 99, 8888 or 9999 that lie above the valid range of numeric items; it keeps −1 in the occupation and industry items as a category for respondents without an occupation. Under this rule 1.0% of cells were imputed, test R^2 was 0.120 and no domain share changed by more than 0.5 percentage points (Table 6 of the main text).

Age adjustment. Natural cubic splines (degree 3, six knots, linear extrapolation) were fitted to the condition count by design-weighted linear regression in the training set only, and applied to both sets. Age band was retained among the candidate determinants so that any residual age structure would be visible rather than silently redistributed to correlated social exposures; it ranked tenth, indicating that a modest amount of age-related structure persists beyond a smooth function of chronological age.

Design-aware validation. Training and test sets contained disjoint primary sampling units, and cross-validation folds preserved the same grouping. Calibrated survey weights were supplied to the fitting procedure of every estimator accepting observation weights, and all reported metrics are weighted. This prevents the optimistic bias that arises when respondents from the same cluster appear in both training and test data.

Sensitivity analyses. The analysis without age residualisation used the raw condition count as outcome and added standardised continuous age to the 151 determinants, keeping age band; the analysis without age band removed that variable from the residualised model. The inclusion-probability analysis fitted the logistic model of Table S9 on all 16,590 respondents, multiplied each included respondent's calibrated weight by the inverse of

the fitted probability, rescaled the product to the original weight total, and repeated the age residualisation, random-forest fit and SHAP attribution with the resulting weights on the same seed-42 split. The missing-data sensitivity added a binary indicator for each determinant above 5% missingness, or dropped those determinants, and repeated the fit and the attribution on the same split. The analysis of younger adults restricted the sample to respondents aged 15 to 44 years before the age residualisation, so that the spline was refitted within that age range. The scripts for all of these analyses are deposited with the main pipeline.

For this revision, the outcome sensitivity recomputed the condition count without the mental health item (p4_6_1_g), without the chronic-pain item (p4_6_1_o) and without both, keeping the analytic sample, the split and the random-forest configuration unchanged; the first-stage spline explained 26.5%, 24.5% and 28.3% of the variance of these counts, respectively. The grouped measures, the survey-weighted averages and the attribution with tuned models (Tables S13 and S14) were computed on the same split, and the scripts are deposited with the main pipeline.
